# Generation and characterization of a novel *gne* Knockout Model in Zebrafish

**DOI:** 10.3389/fcell.2022.976111

**Published:** 2022-10-24

**Authors:** Hagay Livne, Tom Avital, Shmuel Ruppo, Avi Harazi, Stella Mitrani-Rosenbaum, Alon Daya

**Affiliations:** ^1^ Faculty of Marine Sciences, Ruppin Academic Center, Michmoret, Israel; ^2^ Goldyne Savad Institute of Gene Therapy, Hadassah Medical Center, The Faculty of Medicine, The Hebrew University of Jerusalem, Jerusalem, Israel; ^3^ Info-CORE, Bioinformatics Unit of the I-CORE, The Hebrew University of Jerusalem, Jerusalem, Israel

**Keywords:** GNE, GNE myopathy, *gne* KO zebrafish, cell cycle, DNA damage repair pathway, CRISPR/Cas9, skeletal muscle, transcriptomics

## Abstract

GNE Myopathy is a rare, recessively inherited neuromuscular worldwide disorder, caused by a spectrum of bi-allelic mutations in the human *GNE* gene. *GNE* encodes a bi-functional enzyme responsible for the rate-limiting step of sialic acid biosynthesis pathway. However, the process in which *GNE* mutations lead to the development of a muscle pathology is not clear yet. Cellular and mouse models for GNE Myopathy established to date have not been informative. Further, additional GNE functions in muscle have been hypothesized. In these studies, we aimed to investigate gne functions using zebrafish genetic and transgenic models, and characterized them using macroscopic, microscopic, and molecular approaches. We first established transgenic zebrafish lineages expressing the human *GNE* cDNA carrying the M743T mutation, driven by the zebrafish *gne* promoter. These fish developed entirely normally. Then, we generated a *gne* knocked-out (KO) fish using the CRISPR/Cas9 methodology. These fish died 8–10 days post-fertilization (dpf), but a phenotype appeared less than 24 h before death and included progressive body axis curving, deflation of the swim bladder and decreasing movement and heart rate. However, muscle histology uncovered severe defects, already at 5 dpf, with compromised fiber organization. Sialic acid supplementation did not rescue the larvae from this phenotype nor prolonged their lifespan. To have deeper insights into the potential functions of *gne* in zebrafish, RNA sequencing was performed at 3 time points (3, 5, and 7 dpf). Genotype clustering was progressive, with only 5 genes differentially expressed in *gne* KO compared to *gne* WT siblings at 3 dpf. Enrichment analyses of the primary processes affected by the lack of *gne* also at 5 and 7 dpf point to the involvement of cell cycle and DNA damage/repair processes in the *gne* KO zebrafish. Thus, we have established a *gne* KO zebrafish lineage and obtained new insights into *gne* functions. This is the only model where GNE can be related to clear muscle defects, thus the only animal model relevant to GNE Myopathy to date. Further elucidation of *gne* precise mechanism-of-action in these processes could be relevant to GNE Myopathy and allow the identification of novel therapeutic targets.

## Introduction

GNE Myopathy is an adult onset, progressive neuromuscular disease caused by recessive mutations in the *GNE* gene (Eisenberg et al., 2001). To date, more than 260 different mutations have been reported in GNE Myopathy patients worldwide, through the entire 753 aa protein, both in the epimerase and in the kinase coding sequences, and very often one mutation in each domain are combined in the compound heterozygous patients. However, marked GNE deficiency has not been observed in GNE Myopathy patients (Krause et al., 2007). Further, no patient with a double null mutation in GNE could be identified. Although this bifunctional enzyme is well known as the key enzyme in the biosynthesis pathway of sialic acid, no clear relation could yet be established between the mutated enzyme and the pathogenesis of the disease. The obvious hypothesis of impaired sialylation in patients’ muscle cells is still controversial (Hinderlich et al., 2004; Noguchi et al., 2004; Saito et al., 2004; Salama et al., 2005; Broccolini et al., 2010; Sela et al., 2020). The established mouse models are also difficult to interpret since they are either not reliable or do not present any muscle phenotype (Galeano et al., 2007; Malicdan et al., 2007; Ito et al., 2012; Sela et al., 2013; Benyamini et al., 2020). All these failed attempts for generating a GNE Myopathy animal model strongly emphasize that the process by which *GNE* mutations lead to myopathy is certainly not well understood. Several studies by us and others have pointed to possible novel functions of GNE in the cell (Wang et al., 2006; Amsili et al., 2007; Amsili et al., 2008; Harazi et al., 2017).

The lack of both reliable and consistent animal models severely impairs the basic research on GNE function and prevents treatment evaluations. Therefore it is critical to develop novel model systems to comprehensively examine the functions of the GNE protein in muscle, which could eventually explain the pathophysiological downstream defects caused by GNE mutations in GNE Myopathy.

Zebrafish have been recognized as a very potent tool for the study of development and of various human conditions, including neuromuscular diseases. In particular, the *GNE* gene and protein are well conserved between zebrafish and mammals, and especially with the human ortholog. Indeed, we have been able to assess the expression of *gne* in zebrafish and to show its role specifically in muscle morphology and function (Daya et al., 2014). Therefore, the zebrafish system provides a scientific basis and a rationale for generating a loss of function mutant and stable transgenic zebrafish models that could enable the understanding of GNE function in muscle pathogenesis. In these studies we have established transgenic zebrafish lineages carrying the human WT and M743T *GNE* gene as well as a *gne* knock-out fish.

## Materials and methods

### Zebrafish maintenance

Zebrafish (*Danio rerio*) AB and Tupfel long-fin (TL) strains were maintained according to standard laboratory conditions (Westerfield, 1995) in a ZebTEC Zebrafish housing systems (Tecniplast, S.P.A., Italy) at the Faculty of Marine Sciences, Ruppin Academic Center, Michmoret, Israel.

Embryos were kept in Petri dishes in 30 ml sterile 0.5X E2 embryo medium (The zebrafish book 5th Edition, Westerfield, 2007) containing methylene blue (0.3 ppm), in a 28.5°C light-controlled incubator on a 14-h light: 10-h dark cycle.

Prior to any procedure, larvae were anesthetized with 0.04% MS-222 (Tricaine methane-sulfonate, Ethyl 3-aminobenzoate methanesulfonate, Merck). All procedures involving animals were approved by the Volcani Center Animal Care Committee (Approval number IL-18-11–299) and conducted in accordance with the guidelines of the Council for Experiments on Animal Subjects, Ministry of Health, Israel.

### DNA and RNA sampling, extraction and amplification

Genomic DNA was extracted from embryos, whole-larva, larva fin-clip, or mature fish fin-clip in 50 µL lysis buffer (20 mM Tris-HCl, 10 mM (NH_4_)_2_SO_4_, 10 mM KCl, 2 mM MgSO4, 0.1% Triton®-X-100, pH 8.8 @ 25°C) at 95°C for 10 min and transferred to ice for 1 min. Then, 50 μg Proteinase K (V3021, Promega) was added and incubated overnight at 55°C.

Total RNA samples were extracted either from a pool of >10 embryos/larvae, a single larva or from mature fish muscle or brain tissue, by the TRI Reagent^®^ (#T9424, Merck) procedure, and stored at −80°C until further processing. For mature fish tissue, homogenate was done using a RNase-free plastic pestle, and for embryos/larvae, using a 27 G syringe, followed by a 31 G syringe. For transcriptomic analysis, an additional step of ethanol precipitation, was added to the protocol. Isolated RNA was treated with DNase (TURBO DNA-free™ Kit, AM 1907, Thermo-Fisher Scientific, US), and when necessary converted to cDNA by standard procedures (RevertAid First Strand cDNA Synthesis Kit #K1622, Thermo Fisher Scientific, US).

Because the human *GNE* transgene carries the same sequence as the expressed transgenic mRNA (NM_005476.7), all RT reactions included a minus reverse transcriptase control (MRT) to confirm the absence of DNA contamination in the RNA preparations. PCR was performed in a thermal cycler (C1000, Bio-Rad, US).

### Establishment of transgenic lines expressing the human *GNE* gene under the control of the zebrafish *gne* promoter

Two types of transgenic lines were developed using the tol2 transposon system methodology (Urasaki et al., 2006). We have constructed the p(-1.*9gne*:hGNE^M743T/WT^; *insulin*:EGFP) plasmid to carry the zebrafish *gne* promoter sequence (1.946 kb upstream of the zebrafish *gne* gene, Daya et al., 2014), followed by the human *GNE* cDNA (NM_005476.7). These sequences were cloned between the two long terminal repeats (LTR) sites needed for transposase mediated transgenesis. In addition, an EGFP reporter, driven by a 995 bp zebrafish *insulin* promoter sequence (Pisharath et al., 2007), was cloned between the two LTR sites to simplify screening (this promoter is known to confer expression in the pancreas). Two final plasmids were used, one carried the human *GNE* WT sequence and the second one the human *GNE* with the Middle Eastern M743T missense mutation.

Capped transposase mRNA was synthesized *in-vitro* using the mMESSAGE mMACHINE SP6 IVT kit (#AM1340, Thermo Fisher Scientific, US) from a pCS-TP plasmid (kindly provided by Koichi Kawakami), linearized by the NotI restriction enzyme (R0189S, NEB, US). The mRNA mixture was purified (MEGAclear kit, #AM 1908, Thermo Fisher Scientific, US), quantified (NanoDrop™ 2000, Thermo Fisher Scientific, US), and analyzed on a 1% agarose gel.

Microinjection of zebrafish fertilized eggs (250 ng/μL plasmid and 500 ng/μL transposase mRNA in 2 nL volume) was performed using a micromanipulator and a PV830 Pneumatic Pico Pump (World Precision Instruments, Sarasota, FL). The injected embryos were raised to adulthood, and the EGFP expression was monitored using an epifluorescent SZX16 stereomicroscope. Sexually mature F0 EGFP positive fish were crossed with wild-type (WT) fish and the F1 embryos were screened for EGFP expression and sorted. EGFP positive F1 larvae were grown to adulthood and again crossed with WT fish for the establishment of genetically stable F2 transgenic lines. The F2 progeny is expected to be genetically stable, therefore, EGFP positive larvae were sampled for DNA extraction, and a 215 bp PCR product was obtained using primers spanning the position of the M743T founder mutation in exon 12 (see primer sequences, Supplementary Table S1).

### Establishment of a zebrafish *gne* knockout lineage

To knock out the zebrafish *gne* gene, potential CRISPR target sites were considered using the MIT online tool (crispr.mit.edu). The chosen target was selected at an early locus of the gene (in the epimerase domain) at position 395-414 in NM_200883.1. The selected gRNA had no predicted off-targets and contained a HpyCH4IV recognition sequence, for screening purposes. The target sequence (5′-GAATGTCGGGCGCGAGACGT-3′, 20 bp) was copied into a gRNA design template, as described by Hruscha et al. (2013). The reverse complement of the designed gRNA was synthesized as a single-strand DNA oligo (Integrated DNA Technologies Inc.). This DNA oligo served as a template, was hybridized to a T7 oligo, and *in-vitro* transcribed using the MEGAshortscript^™^ T7 transcription kit (#AM1354, Thermo Fisher Scientific, US). The gRNA (2 µg/µL) was co-injected as a complex with the Cas9 endonuclease protein (0.5 µg/µL, PNA Bio Inc.) to the cell-body of one-cell fertilized eggs. DNA was extracted from injected eggs or larvae and amplified as indicated. Typically, 2–5 µL of the PCR reaction were used for digestion with the HpyCH4IV restriction enzyme (#R0619L, New England Biolabs Inc.) or for other screening methods. Sexually mature F0 CRISPR injected fish were crossed with WT fish and the F1 offspring were screened by PCR for CRISPR-directed indels and sorted. CRISPR-positive F1 larvae were grown to adulthood and again crossed with WT fish for the establishment of genetically stable F2 mutated lines. A fin clip was taken from each type of the F2 mutated lines, and positive samples were sequenced.

### Zebrafish *gne* '-4 bp' genotyping

Genotyping of *gne* was done either by PCR (2 reactions per sample), using the *gne* 28F and 427R primers and an additional allele-specific primer (see primer sequences, Supplementary Table S1), or a TaqMan real-time PCR reaction (#4444557, Thermo-fisher, US) with two *gne* allele-specific probes, based on the 4 bp deletion in exon 3, that differ by sequence and by the fluorophore wavelength (WT “JOE”, and mutant “FAM”). Annealing temperature was optimized to 60.5°C in a two-step real-time qPCR reaction (StepOnePlus™ Real-Time PCR System and software, Applied Biosystems, Thermo-fisher, US).

### Macroscopic observations

#### Larvae monitoring

During the first 16 days of development, zebrafish embryos and larvae were monitored using an epifluorescence SZX16 stereomicroscope equipped with a DP73 digital camera (Olympus, Japan). Images were scaled and used for the evaluation of larval development by measuring the body length, body height, eye diameter and somites width using ImageJ software. Statistical analysis was performed using the non-parametric Mann-Whitney/Wilcoxon *U* test (*p*-value < 0.05), and figures were built using excel software.

#### Touch-evoke assay

Zebrafish larva was gently positioned in the middle of a Petri dish with fresh embryo medium and video recorded under a stereomicroscope (According to Sztal et al., 2016). Each larva’s tail was then gently touched using a plastic pipette and the response was documented. The videos were qualitatively analyzed as described in Guzman et al. (2020), using ImageJ software. Briefly, the position of each larva in each frame (every 70 milliseconds) was ranked by receiving a value depending on the evaluated swimming distance post stimuli: Larva that left the test zone received a score of 4; larvae that moved 1/4 of the zone gained a score of 3; larva that moved between 1/4-1/2 of the zone was scored 2; larva that moved between 1/2-1 gained 1; and larvae that moved less than 1/4 of the zone gained a score of 0. The average and standard deviation were calculated for each genotype at 6, 7, and 8 dpf (10 larvae per group). Statistical analysis was performed using the non-parametric Mann-Whitney/Wilcoxon *U* test (*p*-value < 0.05), and figures were built using excel software.

#### Heartbeat rate measurement and analysis

Zebrafish larvae were anesthetized and gently positioned under a stereomicroscope (SZX16, equipped with DP-73 camera, Olympus), then, a 30 s video of the heartbeats was captured. We manually counted the number of heartbeats per video and duplicated the result by 2 to get the number of beats per 1 min. Average and the standard deviation were calculated for *gne* WT and *gne* KO (-4 bp mutated allele) at 5-8 dpf (10 larvae per group). Statistical analysis was done using the non-parametric Mann-Whitney/Wilcoxon *U* test (*p*-value < 0.05), and figures were built using excel software.

#### Larvae locomotor activity tracking and analysis

To quantify the locomotor activity of *gne* ko at 5 and 7 dpf, we used the DanioVision chamber (Noldus, Netherlands). This device was used to monitor zebrafish larvae activity in a 48 well plate, with one larva per well at 28°C. *gne* heterozygote fish were crossed and the offspring were grown in an incubator and then transferred to a 48 well plate which was placed in the chamber. The experiment began with 30 min of acclimation, followed by 1 h of locomotor activity monitoring. Live video was collected using an infrared-sensitive camera and tracking was conducted using the Ethovision XT software (Noldus Information Technology). Following the experiment, larvae were sacrificed and genotyped. Locomotor activity was measured as the mean velocity of each larva for 1 h, post acclimation. Average velocity and standard deviation were calculated for each *gne* genotype group.

Statistical analysis was done with R programming language (version 4.1.3) using the non-parametric Mann-Whitney/Wilcoxon *U* test (*p*-value < 0.05), with an FDR adjusted *p*-value of 0.01.

#### Birefringence assay

To assess muscle fibers integrity, we used the Birefringence assay. Zebrafish larvae were anesthetized and gently positioned on the side in 3% methyl cellulose (#M0387, Merck) on a polarizer filter. The top analyzer filter (SZX2-AN, Olympus, Japan) was mounted on the stereomicroscope lens (SZX16, equipped with DP-73 camera by Olympus, Japan). The analyzer filter was rotated until a dark background appeared, and the organized skeletal muscle appeared bright in WT larvae. Pictures were taken as gray-scale photos. Then, using ImageJ software, images were scaled, and the average intensity was calculated per muscle area. Statistical analysis was done using the non-parametric Mann-Whitney Wilcoxon *U* test (*p*-value < 0.05), and figures were built using excel software.

### Microscopic analysis

#### Histology

Zebrafish larvae at the first 10 dpf were anesthetized, fin-clipped, genotyped, and then fixed in ice-cold 4% PFA solution (formaldehyde 4% buffered, PH 7.2–7.4, Bio-Lab, Israel) for 24 h (rocker, 4°C), then washed 5 times for 10 min (rocker, RT) with PBST (1x PBS, 0.1% tween20 (#P7949, Merck) and used for downstream procedures. Fixed larva at 5 and 8 dpf were dehydrated in EtOH serial dilutions (50%, 70 and 95% in DDW, 5 min each) and embedded in JB4 resin (JB-4^®^ Mini Embedding Kit, #22507-1, Polysciences, Inc.) according to manufacturer’s instructions. 5–7 µm sections were cut with LKB 8800 Ultratome III microtome and manually transferred to microscope slides and dried by placing the slides on a 50°C Hot plate for ∼30 s. Eventually, slides were stained with Hematoxylin and Eosin (H&E) according to the manufacturer’s instructions. Slides were imaged using a brightfield Olympus BX41 microscope, equipped with a DP72 camera (Olympus, Japan).

#### Whole-mount larvae F-actin staining

Fixed larva at 5 and 8 dpf were treated with PBS-tritonX-100 (1x PBS, 2% triton (#T8787, Merck) for 90 min (Rocker, RT), then stained over-night (Rocker, RT) with rhodamine-labeled phalloidin (5 assays/ml in PBS-tritonX, #R415, Thermo Fisher Scientific, US) and washed 3 times for 20 min with PBST (Rocker, RT).

#### Whole-mount larvae immunostaining

Fixed larvae at 5 and 8 dpf were digested in 1 mg/ml collagenase (#C9891, Merck) for 90 min (rocker, RT) and then washed twice with PBST for 10 min (rocker, RT). Larvae were permeabilized with cold acetone for 10 min in glass tubes, then washed twice with PBST again, followed by blocking with 10% goat serum in BDP (0.1% BSA, 1% DMSO- PBS). Immunostaining was performed with the following primary antibodies: 1/5 anti-MyHC (F59, DSHB, USA), 1/300 anti-α-actinin (clone EA-53, #A7811), 1/300 anti-α-actin (Ac1-20.4.2, Progen, Germany). The larvae were incubated overnight with the primary antibody (rocker, 4°C) and washed for 20 min 3 times in PBST. Larvae were then incubated with fluorescence-labeled secondary antibody (1/200 Alexa Fluor^®^ conjugated goat anti-mouse IgG antibody, # A-11017, Thermo Fisher Scientific, US) for 1 h at room temperature and washed for 30 min 3 times in PBST (rocker, RT). For confocal microscopy, zebrafish larvae were mounted using 1.8% 'low melt' agarose gel to allow proper positioning. Confocal imaging of fixed larvae subjected to F-actin staining/immunostaining was performed using either an Olympus Fluoview 300 confocal microscope configured on an IX70 inverted microscope with a DP70 digital camera (Olympus, Japan) or an LSM780 upright confocal microscope (Zeiss, Germany). Imaging was conducted in the Core Research Facility (CRF) of the Faculty of Medicine, The Hebrew University-Hadassah Medical School, at Jerusalem, or at the Leslie and Susan Gonda Multidisciplinary Brain Research Center, Bar-Ilan University. Image analysis was performed using the ImageJ software.

### Maternal inheritance analysis

To determine whether maternal *gne* mRNA is present during the first week of development, a custom TaqMan qPCR was designed to amplify zebrafish *gne* mRNA. To distinguish between the WT and the mutant *gne* alleles, two allele-specific probes were designed, based on the 4 bp deletion in exon 3. The forward primer binds exons 2-3 junction, to allow only cDNA amplification, and the reverse primer binds position 170 of exon 3. In addition, the probes also differed by the fluorophore type ("JOE"-WT, “FAM"-KO) (see primer sequences, Supplementary Table S1).

RNA was extracted from offspring pools of in-crossed WT and *gne* heterozygotes at 1 and 3 hpf (hours post fertilization, *n* = 10). cDNA was synthesized and used as a template for the TaqMan PCR reactions. The *eef1a1b* gene was used as reference (primer sequences, Supplementary Table S1), and a 3 dpf zebrafish *gne* heterozygote cDNA was the calibrator. Calculations were done using the 2^–∆∆Ct^ method for each probe independently.

### Sialic acid rescue experiment

Embryos were collected, after natural spawning by zebrafish *gne* ^+/-^ in-cross, to 0.5X E2 medium. At ∼2 h post fertilization, the chorion was manually punctured, and the offspring were randomly separated to control and experimental groups. Sialic acid (#A0812, Merck, Germany) was added to a sterile 0.5X E2 medium at final concentrations of 200, 400, and 800 µM. The experimental group’s medium contained sialic acid, and the medium was replaced twice a day. At 1 dpf, larvae were manually dechorionated in both groups. At 7 dpf, larvae were separated into groups of 15, the medium was replaced once a day, and the final volume increased from 30 to 60 ml. Up to 16 dpf, we monitored the larvae and sampled phenotypic larvae for genotyping at each point, and fin-clipped all survivors at 16 dpf for genotyping. Mortality and phenotype appearance were documented and were eventually crossed with the genotype data (methods, Zebrafish *gne* '-4bp' genotyping by TaqMan qPCR) of the sampled larvae.

### Zebrafish *gne* KO transcriptomics

We sampled the 3 *gne* genotypes, wild-type (WT), heterozygote (HT) and knockout (KO), at 3 time points (3, 5, and 7 dpf) with 3 biological repeats (each repeat contained 20 larvae). Therefore, we repeated the same in-cross of zebrafish *gne* ^+/-^ (−4 bp deletion). Eventually, each larva was genotyped by PCR, and 20 larvae for each time-point, and each genotype were pooled together, homogenized, and RNA was extracted as described above. RNA was analyzed for RNA Integrity Number (RIN) using the TapeStation System and software (Agilent Technologies, Inc.). All samples were sent for RNA sequencing (The Center for Genomic Technologies, the Hebrew University and Hadassah Medical School, Jerusalem, Israel). We chose a one-sided run using the KAPA Stranded mRNA-Seq Kit (KK8420, Roche, Sweden), using Illumina NextSeq 500 system.

All the processing, Bioinformatics, and statistical analysis were performed at the Info-CORE, Bioinformatics Unit of the I-CORE at The Hebrew University of Jerusalem, Jerusalem, Israel.

Normalization and differential expression analysis were done with the DESeq2 package (R programming). Normalized counts were used for several quality control assays, such as counts distributions and principal component analysis, that were calculated and visualized in R. Differential expression was calculated with a design that used two different statistical tests: 1) The first was pairwise comparisons (using the default Wald test), comparing each pair of genotypes for every time point, and every pair of time points for each genotype. Processed FASTQ files were aligned to the zebrafish transcriptome and genome with TopHat, and quantification was done using HTSeq-count. The genome version used was GRCz11, with annotations from Ensembl release 95. Normalization and differential expression analysis were done in R, using the DESeq2 package. Significance threshold was taken as padj <0.1. All data and details of the processing have been deposited at GEO (https://www.ncbi.nlm.nih.gov/geo/) and are available under GEO accession number GSE207593.

### Gene enrichment analysis

Lists of up/down-regulated differentially expressed genes between *gne* KO to *gne* WT at each time point were converted to a list of known human orthologues. The list of human orthologs was the input for gene enrichment analysis using GeneAnalytics (https://geneanalytics.genecards.org/) and IPA software (Qiagen, Germany).

### Gene set enrichment analysis

The input for GSEA analysis is the complete expression pattern (All transcripts). GSEA (https://www.gsea-msigdb.org/gsea/index.jsp) was done to determine whether an *a priori* defined set of genes shows statistically significant, concordant differences between *gne* WT to *gne* KO at all-time points.

### Gene expression validation using TaqMan assays

Validation was done by using the same RNA samples used for RNA sequencing. cDNA was synthesized (as previously described) for *gne* KO and *gne* WT at 3 and 7 dpf with random primers. We selected 8 genes for this purpose, and used verified TaqMan-based assays for each one of those genes (Thermo-Fisher Scientific, US, see primer sequences, Supplementary Table S1). The *eef1a1a* gene was used as a reference for normalization. The calibrator was the WT sample at each time point. Calculations were done using the 2^–∆∆Ct^ method for each probe independently. We compared these fold-change values to the values obtained in the transcriptomic analysis. To evaluate concordance in gene expression intensities between RNA-seq and qPCR, we first calculated the expression correlation between normalized RT-qPCR Cq-values and log transformed RNA-seq expression values. Pearson correlation analyses and linear regression between the RNA-seq and RT-qPCR fold-change results were performed using (GraphPad Software, Prism 9). Statistical analysis was done using the non-parametric Mann-Whitney Wilcoxon *U* test (*p*-value < 0.05 was considered statistically significant).

## Results

### Generation of transgenic human GNE zebrafish lineages

**FIGURE 1:**
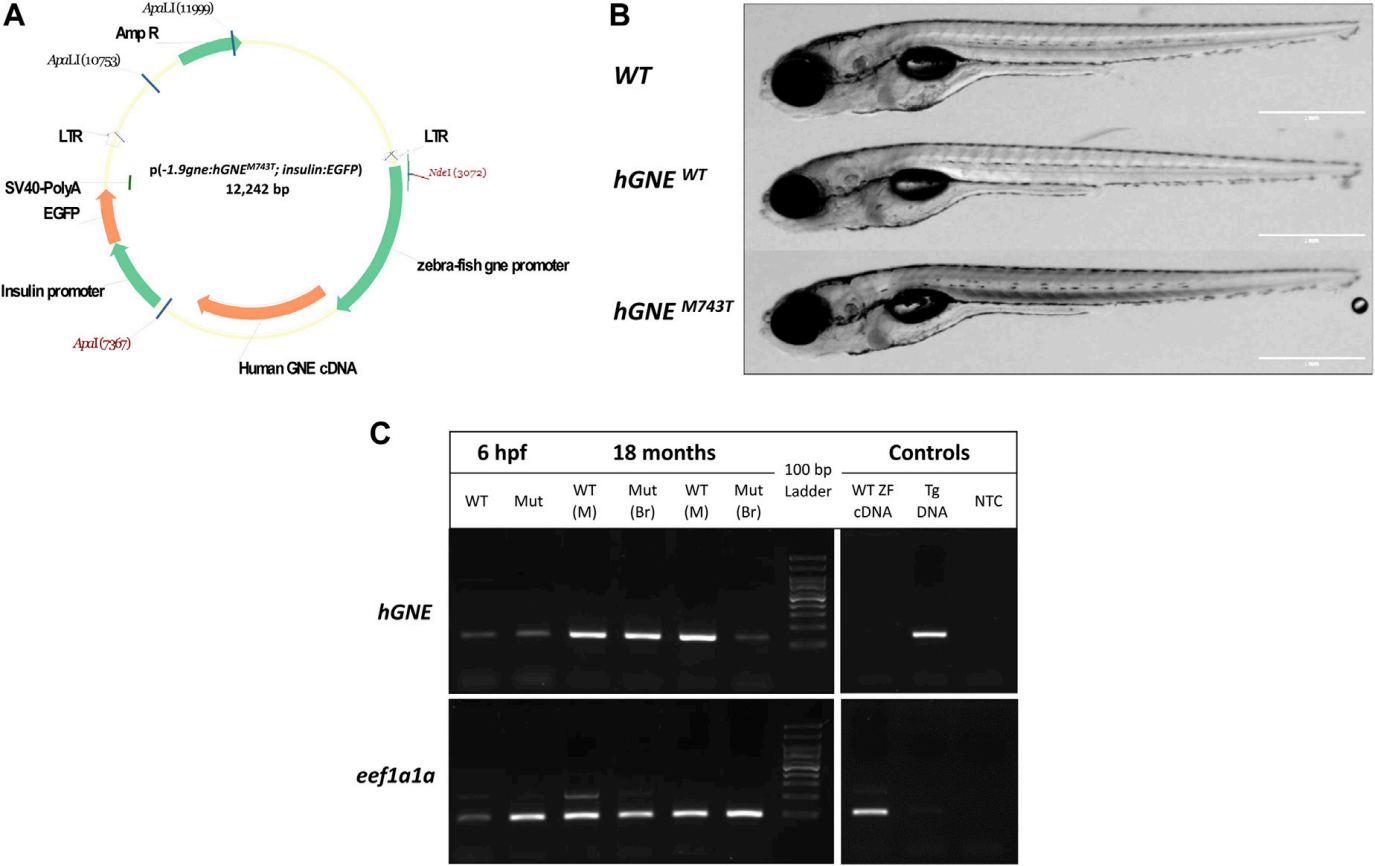
Generation of *hGNE* ^M743T/WT^ transgenic lines. **(A)** A schematic representation of the p(-1.9*gne*:hGNE^M743T/WT^; *insulin*:EGFP) plasmid. Genes, promoters, LTRs, polyadenylation signal and restriction sites are indicated. **(B)** The *hGNE* transgenic lines develop normally. The figure presents brightfield images of a WT (top), *hGNE* ^*WT*^ transgenic (middle), and *hGNE* ^*M743T*^ transgenic (bottom) larvae at 8 dpf. Scale bar = 1 mm. **(C)** RT-PCR analysis of *hGNE* mRNA expression in *hGNE* ^M743T^ and *hGNE* ^WT^ transgenic lines. Agarose gel electrophoresis of RT-PCR amplification products of the *hGNE* gene (top) and *eef1a1a* (bottom) as the internal control. Both the *hGNE* ^M743T^ (Mut) and *hGNE* ^WT^ (WT) cDNA samples show the expected amplification in all tested time points and tissues. Non-transgenic WT zebrafish cDNA (WT ZF cDNA) and genomic DNA from transgenic fish (Tg DNA) served as controls for each target. (NTC) no template control, (M) Muscle, (Br) Brain tissue.

Using the Tol2 transposon system, two transgenic zebrafish lineages were established by introducing a construct carrying either the human WT or the *GNE M743T* mutated cDNA 2212 bp sequence (derived from NM_005476.5), both driven by the zebrafish *gne* 1.9 kb promoter (Daya et al., 2014). The *GNE* sequences were cloned into the pTol2(*insulin*:EGFP) backbone plasmid (Figure 1A).

The use of the zebrafish *insulin* promoter in the microinjected plasmid resulted in EGFP expression in the zebrafish pancreas, to facilitate screening. F0 EGFP positive embryos were grown to adulthood and crossed with wild-type zebrafish in order to find germline transmitters, and for the establishment of F1 and then F2 stable transgenic zebrafish lines. F2 progeny was sequenced to assess the presence of the zebrafish *gne* promoter and *hGNE* cDNA transgene. F2 offspring of the two types of transgenic lines (*hGNE* M743T and *hGNE* wild-type) were monitored and found to develop normally, were fertile and had a normal lifespan (Figure 1B).

Expression of either wild-type or M743T mutated human *GNE* was verified by RT-PCR from 6 hpf embryos till 18 month old fish. (Figure 1C). Therefore, it can be concluded that WT *hGNE* and M743T *hGNE* expression do not impair zebrafish development and lifespan.

### Generation of *gne* knockout zebrafish lines using CRISPR/Cas9

**FIGURE 2:**
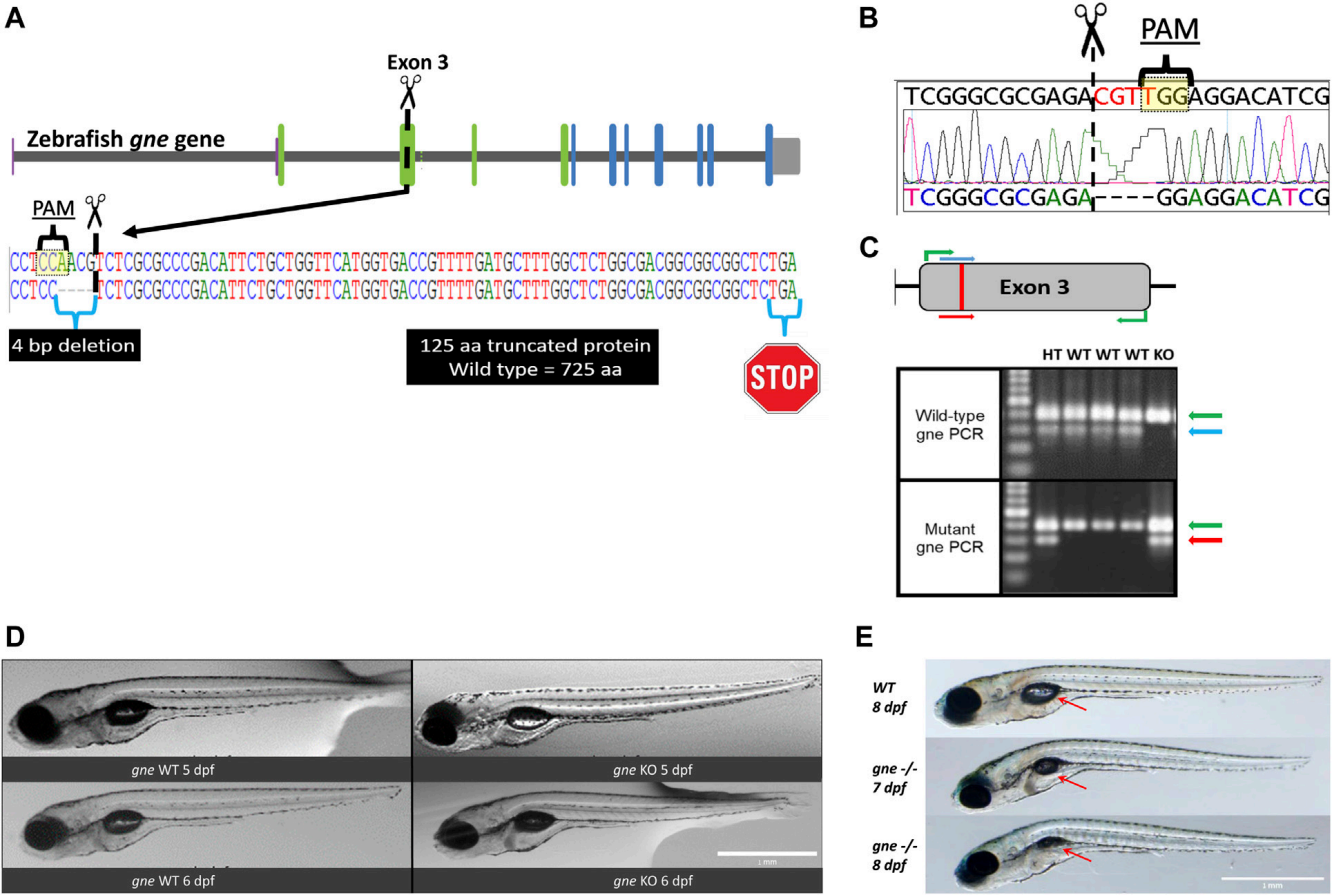
Generation and development of the *gne* knockout zebrafish. **(A)** Schematic representation of CRISPR/Cas9 mutagenesis in the zebrafish *gne* gene, exon 3. A diagram of the *gne* gene containing 12 exons, represented by green (epimerase domain) and blue (kinase domain) rectangles, and the UTRs. The PAM sequence is specified, position of the generated 4 bp deletion site is indicated by scissors symbol and the putative premature termination codon is depicted by a stop symbol. **(B)** Sanger sequencing chromatogram of *gne* KO larvae (bottom sequence) indicating a 4 bp deletion in *gne* exon 3. The PAM sequence and the double-strand break position (scissors) are indicated. **(C)** Zebrafish *gne* alleles PCR genotyping. The top panel represents a schematic view of the primers’ position in each PCR reaction. Wild-type specific PCR is presented on the top gel, and mutant specific on the bottom gel. Green arrows represent a 399 bp amplicon of exon 3. Blue arrows represent a 300 bp wild-type (WT) specific amplicon, red arrows represent the mutant (KO) specific amplicon. Heterozygotes (HT) show both allele-specific amplicons. 100 bp DNA marker was loaded on the left lanes of each 1.5% agarose gel. **(D)** Zebrafish *gne* KO larvae exhibit a normal phenotype at 5 and 6 dpf. *gne* genotype and age are indicated at the bottom of each image. Scale bar = 1 mm. **(E)** Zebrafish *gne* KO phenotype. Zebrafish *gne* KO larvae present deflation of the swim bladder and progressive curving of the body at 7 and 8 dpf, compared to WT zebrafish at 8 dpf. Red arrows indicate the swim bladder. Scale bar = 1 mm.

Next, we generated an endogenous *gne* knockout zebrafish, using the CRISPR/Cas9 system. The guide RNA was designed to target the minus strand of exon 3 of the zebrafish *gne* gene (position 395-414, NM_200883.1) (Figure 2A). F1 progeny screening showed various sequence alterations at the targeted *gne* locus. An F1 mutant line carrying a 4 bp deletion (henceforth referred to as *gne* ^*−/−*^ or *gne* KO) was selected to obtain homozygous mutants for *gne* KO characterization (Figures 2A,B). This mutation results in a frameshift, leading to an early stop codon and subsequently a 125 amino acid truncated protein. F1 heterozygotes for the mutated allele were identified by allele-specific PCR (Figure 2C), grown to adulthood and out-crossed with WT fish for the F2 generation. We repeated these crosses to reduce background mutations and established an F4 progeny. The mutated *gne* transcripts were confirmed by RT-PCR and sequencing (Figure 2B).

### *gne* ^*−/−*^ larvae have initial normal gross morphology and activity but die by 10 dpf

**FIGURE 3:**
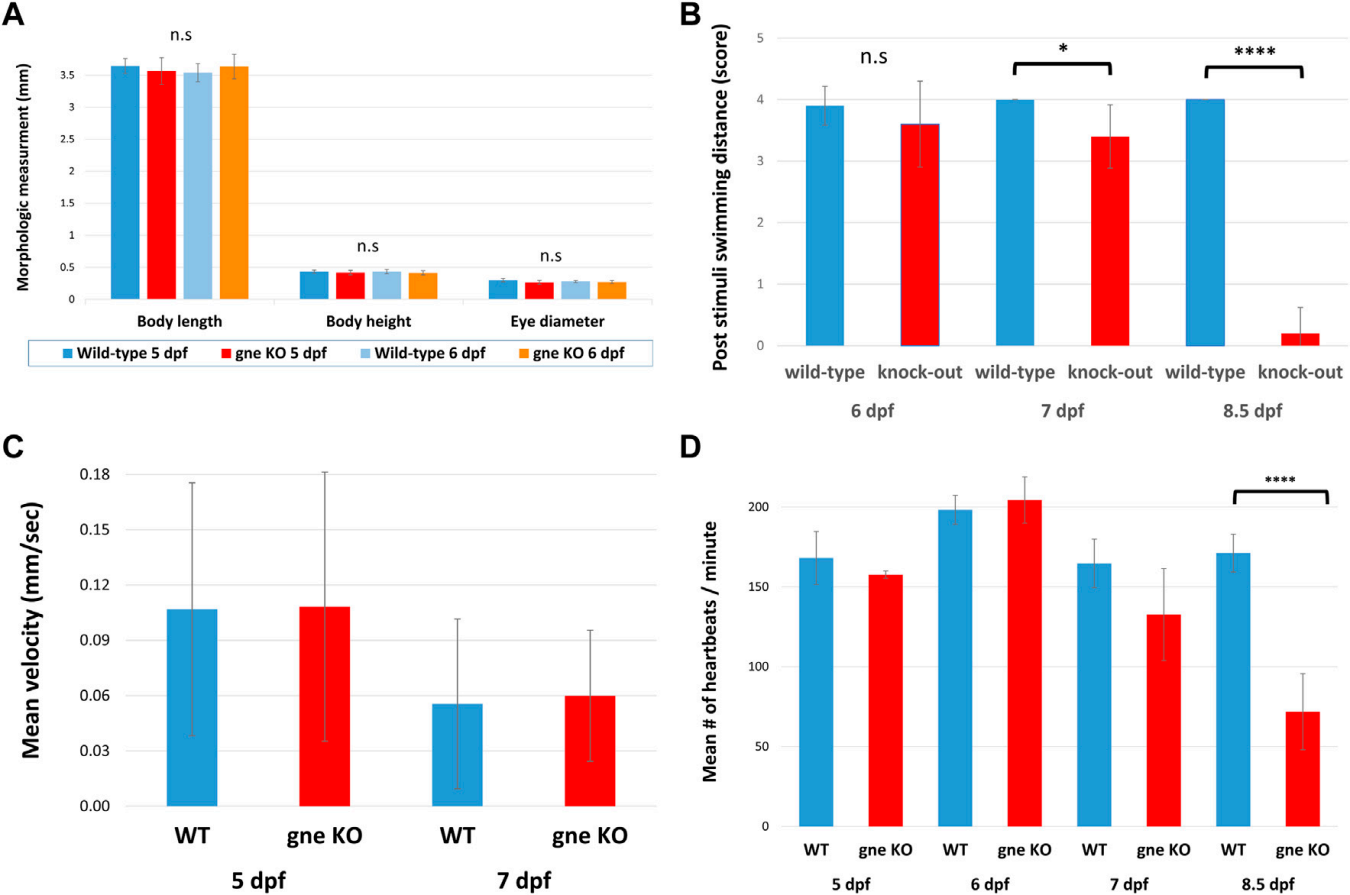
Structural and functional characterization of the *gne* knockout larvae. **(A)** Analysis of morphologic measurements (body length, body height, eye diameter) in *gne* ^*−/−*^ mutants and WT larvae at 5 and 6 dpf did not show any statistically significant differences (Mann-Whitney Wilcoxon test, *n* = 40, *p* > 0.05). **(B)** *gne* KO larvae display reduced response to tactile stimuli in 'Touch-evoke assay'. Quantification of the assay revealed that *gne* KO larvae swim significantly less distance, compared to WT larvae, following tactile stimuli at 7 and 8.5 dpf (Mann-Whitney Wilcoxon test, *n* = 60). **(C)** Locomotor activity is not affected in *gne* KO larvae relative to their WT sibling. The mean velocity was calculated for each larva for 60 min, then averaged for each *gne* genotype at 5 and 7 dpf. (Mann-Whitney Wilcoxon test, *n* = 133). **(D)** Zebrafish *gne* KO heartbeat rate is significantly reduced only at 8.5 dpf (Mann-Whitney Wilcoxon test, *n* = 80). Significant differences are indicated by *p < 0.01, ****p < 0.00001, standard errors are presented.

*gne* ^*−/−*^ mutants appeared to develop normally and were phenotypically indistinguishable from their wild-type siblings at 6 dpf (Figure 2D). Analysis of morphologic measurements did not show any statistically significant differences in body length, body height or eye diameter between *gne* ^*−/−*^ mutants and WT larvae at 5 and 6 dpf (Figure 3A). Somite width measurements of *gne* ^*−/−*^ mutants did not significantly differ from wild-type larvae at 6 dpf (Supplementary Figure S1).

However, starting from 7 to 8 dpf, the posterior swimming bladder of *gne* ^***−/−***^ homozygotes progressively deflated, and their body axis gradually began to curve (Figure 2E). Approximately 20 h after the appearance of this phenotype, the *gne* KO larvae stopped moving and eventually died by 8–10 dpf.

To assess their neuromuscular function, we performed a touch-evoke escape assay at 6, 7 and 8.5 dpf. Indeed, zebrafish *gne* KO larvae showed a significantly reduced response to the tactile stimuli at 7 and 8.5 dpf, but not at 6 dpf (Figure 3B). Further analysis of their locomotor activity using the DanioVision system revealed no significant difference in mean velocity between *gne* KO and *gne* WT larvae at 5 dpf and 7 dpf (Figure 3C).

Therefore, it seems that the lack of *gne* does not affect locomotor activity before 7 dpf.

Similarly, monitoring of the heartbeat rate in *gne* ^***−/−***^ larvae showed a slight reduction compared to their wild-type siblings at 7 dpf, which worsened and was statistically significant only at 8.5 dpf, just before they died (Figure 3D). These experiments were conducted in a blinded manner on siblings from *gne* ^***+/–***^ in-cross, which were genotyped only after the completion of the assay.

### Muscle integrity is disrupted in *gne* KO larvae after 7 dpf

**FIGURE 4:**
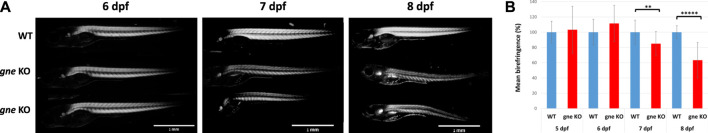
*gne* KO results in skeletal muscle defects which appear at 7 dpf. **(A)** A reduction in muscle birefringence is observed in *gne* KO larvae at 7 dpf, compared to WT sibling, and correlates to the severity of the phenotype at 8 dpf. **(B)** Quantification of the mean birefringence revealed a significant reduction in birefringence at 7 dpf, and highly significant at 8 dpf, compared to WT sibling controls (Mann-Whitney Wilcoxon test, *n* = 60). Significant differences are indicated by *p < 0.05, **p < 0.01, ****p < 0.00001, standard errors are presented.

To obtain data on muscle integrity, we first conducted a muscle birefringence assay. Measurements of polarized light diffraction while passing through the pseudo-crystalline array of the muscle sarcomeres enables the quantification of the amount of organized myofibril in the trunk musculature (Berger et al., 2012). No obvious differences were observed in muscle birefringence between *gne* WT and *gne* KO up to 6 dpf (Figures 4A,B).

However, at 7 dpf, concomitantly with *gne* KO phenotype onset, a significant reduction in birefringence became obvious in the *gne* KO larvae, which worsened at 8 dpf, just prior to their death. Importantly, the birefringence of the homozygous mutants appeared uniformly reduced throughout the trunk musculature, as seen in mutants in which there is a defect in myofibril organization (Berger et al., 2012; Berger et al., 2022), as opposed to a patchy pattern of birefringence which indicates loss of whole myofibers due to detachment and degeneration (Kawahara et al., 2011).

**FIGURE 5:**
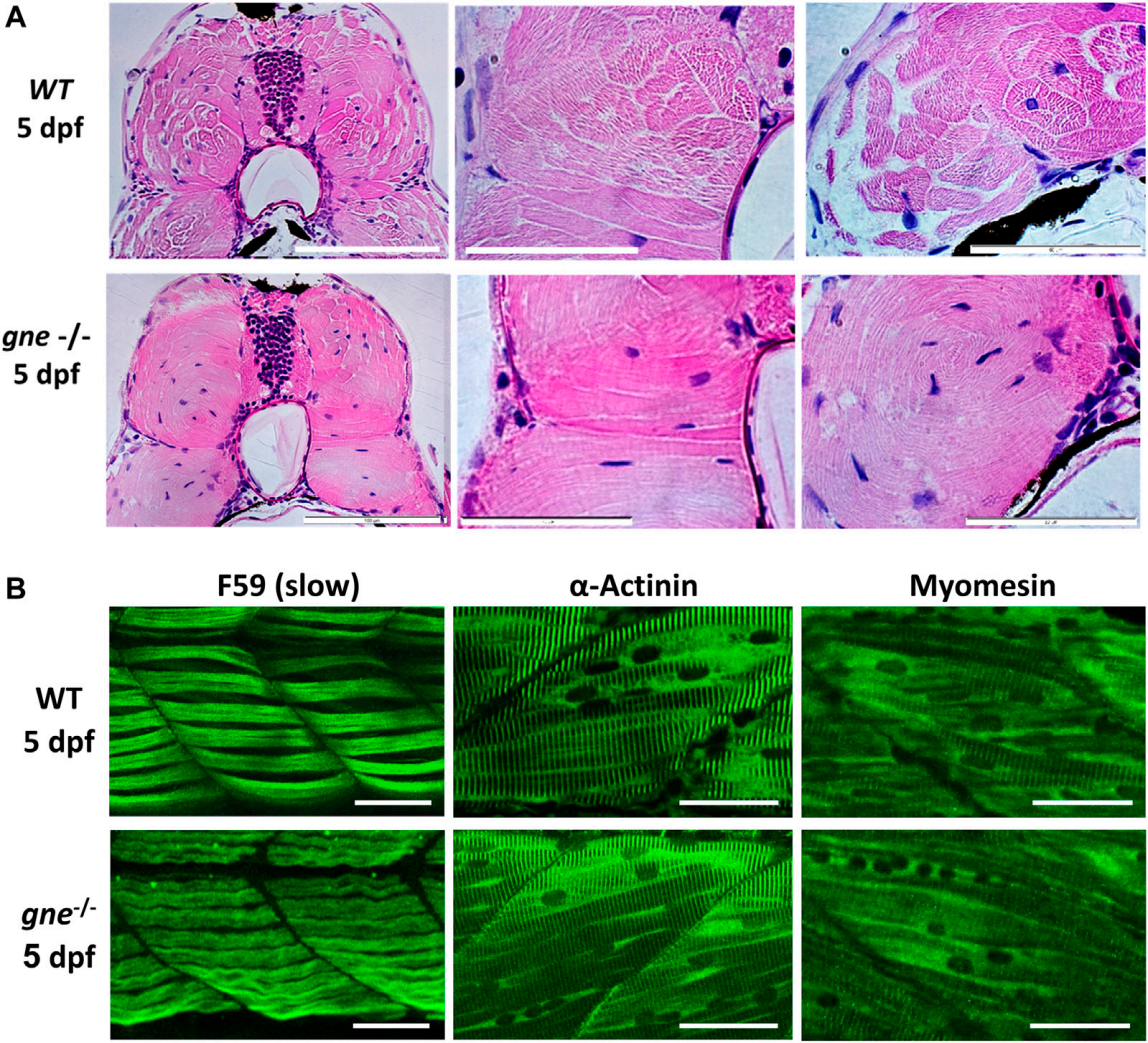
*gne* KO larvae present disorganization of muscle fibers at 5 dpf. **(A)** H&E staining of muscle cross-sections of *gne* WT (top) and *gne* KO (bottom) larvae display abnormally organized myofibers with undefined fiber bundles. Scale bar = 100 µm (left), 50 µm (middle and right). **(B)** Confocal imaging of whole-mount immunostained larvae at 5 dpf by *gne* genotype. *gne* KO exhibits a slightly wavy pattern of the slow muscle fibers (F59) and structurally normal fast fibers (stained with Actinin and Myomesin) with proper localization. Scale bar = 50 µm.

This suggests that muscle development is not affected initially in *gne* KO larvae, but muscle structure could deteriorate around 7 dpf onward. However, a better resolution facilitated by muscle histology of these *gne* KO larvae confirmed abnormally organized myofibers with less defined fiber bundles already at 5 dpf (Figure 5A). Defects were also seen in brain and eye structures: we observed differences in layers of the cell types forming the retina of *gne* KO larvae at 5 dpf. The brain of *gne* KO exhibits an impaired shape, but only at 8 dpf (Supplementary Figures S2, S3).

Whole-mount immunostaining of 5 dpf *gne* KO using F59 ab revealed a mild waviness of the slow-muscle fibers. In contrast, this phenotype was absent in the fast-muscle fibers stained with actinin and myomesin ab, which presented proper organization and localization in the muscle tissue (Figure 5B).

**FIGURE 6:**
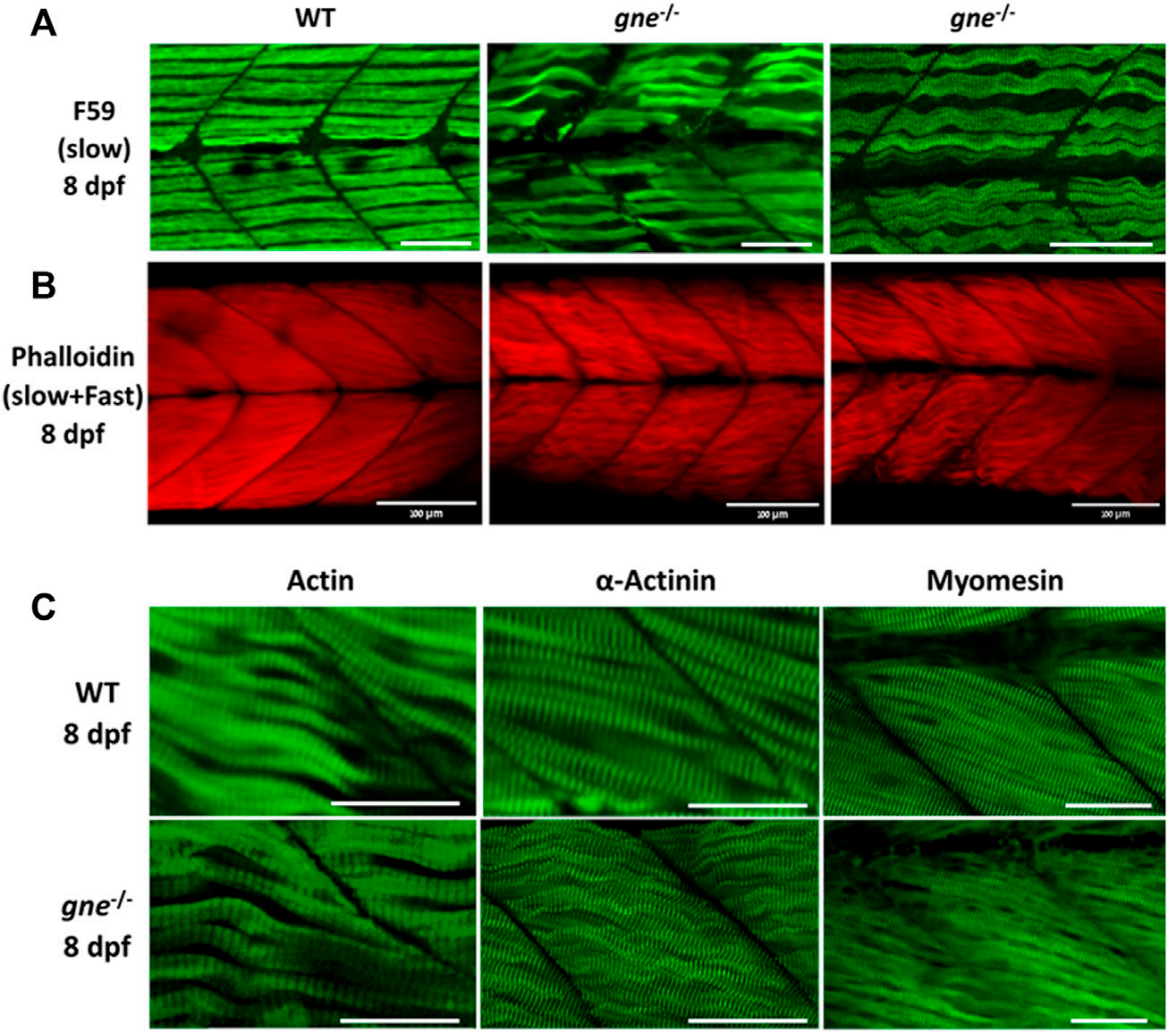
*gne* KO larvae present disorganized slow and fast muscle fibers at 8 dpf. **(A)** Confocal imaging of F-actin stained *gne* KO muscle at 8 dpf. *gne* KO larvae show an abnormal Phalloidin staining pattern relative to the WT control at 8 dpf. Scale bar = 100 µm. **(B)** Confocal imaging of whole-mount immunostained larvae at 8 dpf by *gne* genotype. Zebrafish *gne* KO larvae exhibit impaired organization of the slow muscle fibers, stained with F59 antibody. Scale bar = 50 µm. **(C)** Confocal imaging of whole-mount immunostained larvae at 8 dpf by *gne* genotype. *gne* KO larvae exhibit a wavy pattern of the fast muscle fibers, and correct localization of Actin, Actinin, and Myomesin. Scale bar = 50 µm.

At 8 dpf however, immunostaining of *gne* KO larvae muscle visualizing F-actin, myosin heavy chain in slow fibers, actinin and myomesin proteins, all showed a loose organization of the fibers and a wavy structure, compared to WT (Figure 6), although their myofibrils still showed typical striation (Figure 6C). Birefringence, histology and immunostaining findings point to muscle organization deterioration of both slow and fast fibers as early as 5 dpf.

### Maternal inheritance of *gne* mRNA is not effective at least from 3 dpf

**FIGURE 7:**
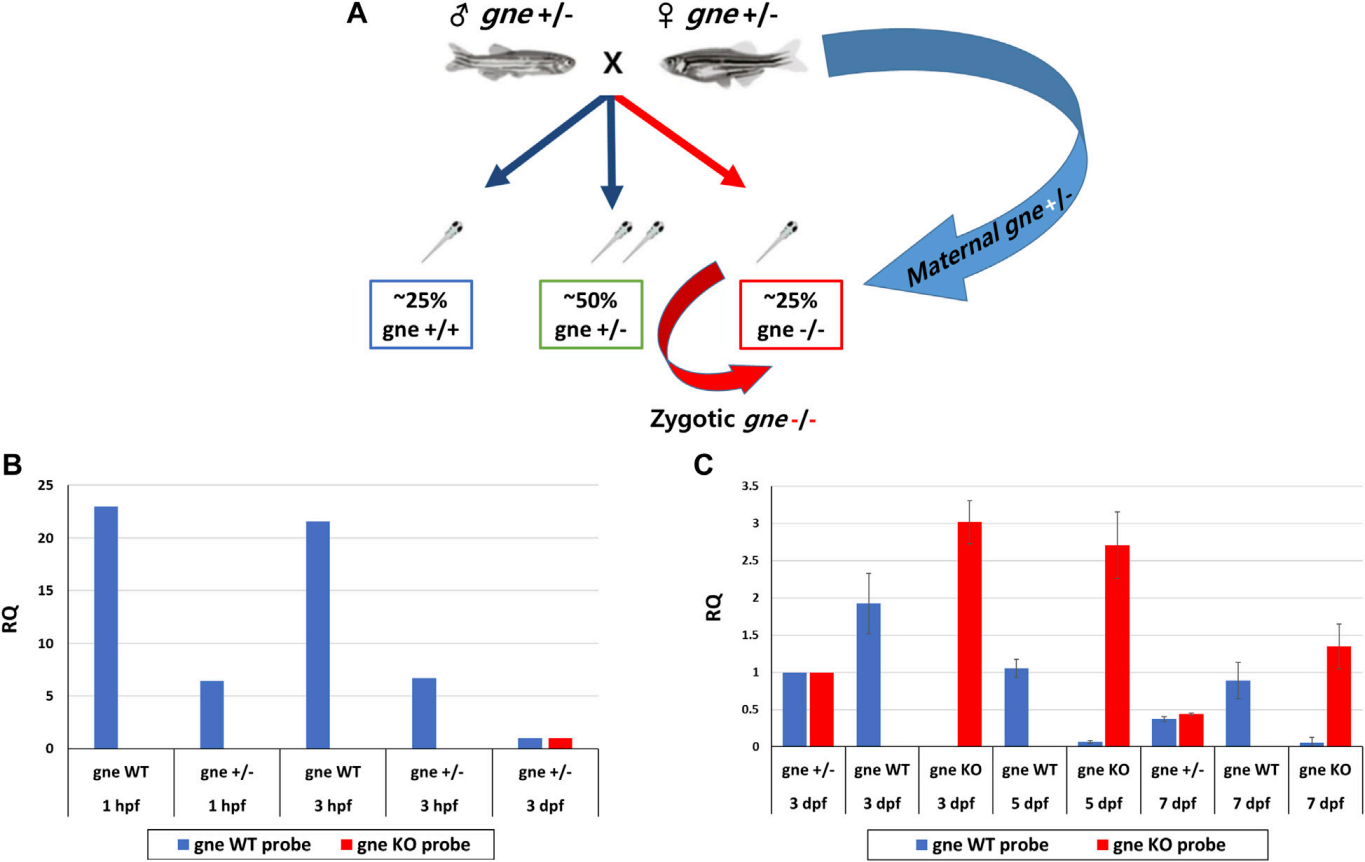
Maternal and zygotic expression of *gne* WT and *gne* KO alleles. **(A)** *gne* maternal inheritance. *gne* ^+/-^ in-cross results in 3 genotypic groups. *gne* KO embryos are predicted to receive both *gne* ^+^ and *gne* ^−^ maternal transcripts (blue arrow), but the zygotic transcription will only transcribe *gne* ^−^ alleles (red arrow). **(B)** Maternal expression of *gne* WT and *gne* mutant allele at 1 and 3 hpf embryos. The relative quantification (RQ) values of *gne* WT (blue bars) and *gne* mutant alleles (red bars) are shown by genotype and age. The WT allele was highly expressed in the WT samples and was lower in *gne* ^+/-^ offspring at 1 and 3 hpf. No amplification was obtained for the gne KO allele. cDNA sample of *gne* HT at 3 dpf was used as a calibrator for all reactions since it expresses both the WT and the KO alleles. **(C)** The relative expression of *gne* WT and *gne* KO alleles at 3, 5, and 7 dpf. The WT allele was highly expressed in the WT samples but was not found in *gne* KO at all-time points, and the mutated allele showed the opposite behavior.

Since phenotype and death did not appear progressively but rather suddenly, we investigated whether the somewhat “delayed” phenotype of *gne* KO larvae up to day 7 post fertilization was a result of the perdurance of maternally deposited WT *gne* proteins or translation products of maternal transcripts up to this stage, as illustrated in Figure 7A.

Maternal inheritance of *gne* mRNA and protein in zebrafish is known to be the only *gne* expression detected until 8 hpf, when zygotic *gne* expression begins (Daya et al., 2014). Maternal *gne* expression was examined by a custom real-time TaqMan PCR reaction with allele-specific probe sets designed to detect either the WT, thus maternally expressed, *gne* mRNA, or the zygotic *gne* mRNA, expressed from the mutated (4 bp deletion) gene. The 2 probes differed by sequence and fluorophore.

Offspring from *gne* ^+/-^ in-cross (or WT in-cross offspring as control) were pooled (*n* = 20) at 1 and 3 h post-fertilization and sampled for RNA extraction. At 3, 5 and 7 dpf, larvae were pooled separately by genotype. At 1hpf and 3hpf, the *gne* WT allele is highly expressed in the WT embryos, and also in a pool of *gne* ^+/-^ crossing offspring, although at a lower level, as expected from maternal transmission (Figure 7B). Mutant allele expression was undetectable in the wild-type control but also, more interestingly, in *gne* ^+/-^ offspring (Figure 7B). Therefore, it seems that the *gne* KO mRNA is not maternally inherited even at these very early stages. At further stages of development, at 3, 5 and 7 dpf, wild-type allele expression was found in all *gne*-wild-type samples, and also in *gne* ^+/-^, at about half level, as expected (Figure 7C). Wild-type allele expression was undetectable in *gne* KO embryos at these stages. In contrast, expression of the *gne* KO allele was found in all *gne* KO larvae, and mRNA level was much higher than in the *gne* ^+/-^ embryos. From these results, we can conclude that maternal *gne* KO mRNA is either not inherited or cannot be detected. More importantly, *gne* WT mRNA is maternally inherited and expressed only at the first stages of the larvae development, but entirely decays by 3 dpf.

### Sialic acid does not rescue *gne* KO larvae from death at 8–10 dpf

Maternally inherited WT *gne* is not effective at least from day 3 post fertilization, but it could still be that the embryos survive till day 8–10 because they are “protected” by the sialic acid synthesized through the maternally WT *gne* inherited at earlier stages. In this case, supplementation of sialic acid should rescue or at least delay death beyond days 8–10.

**FIGURE 8:**
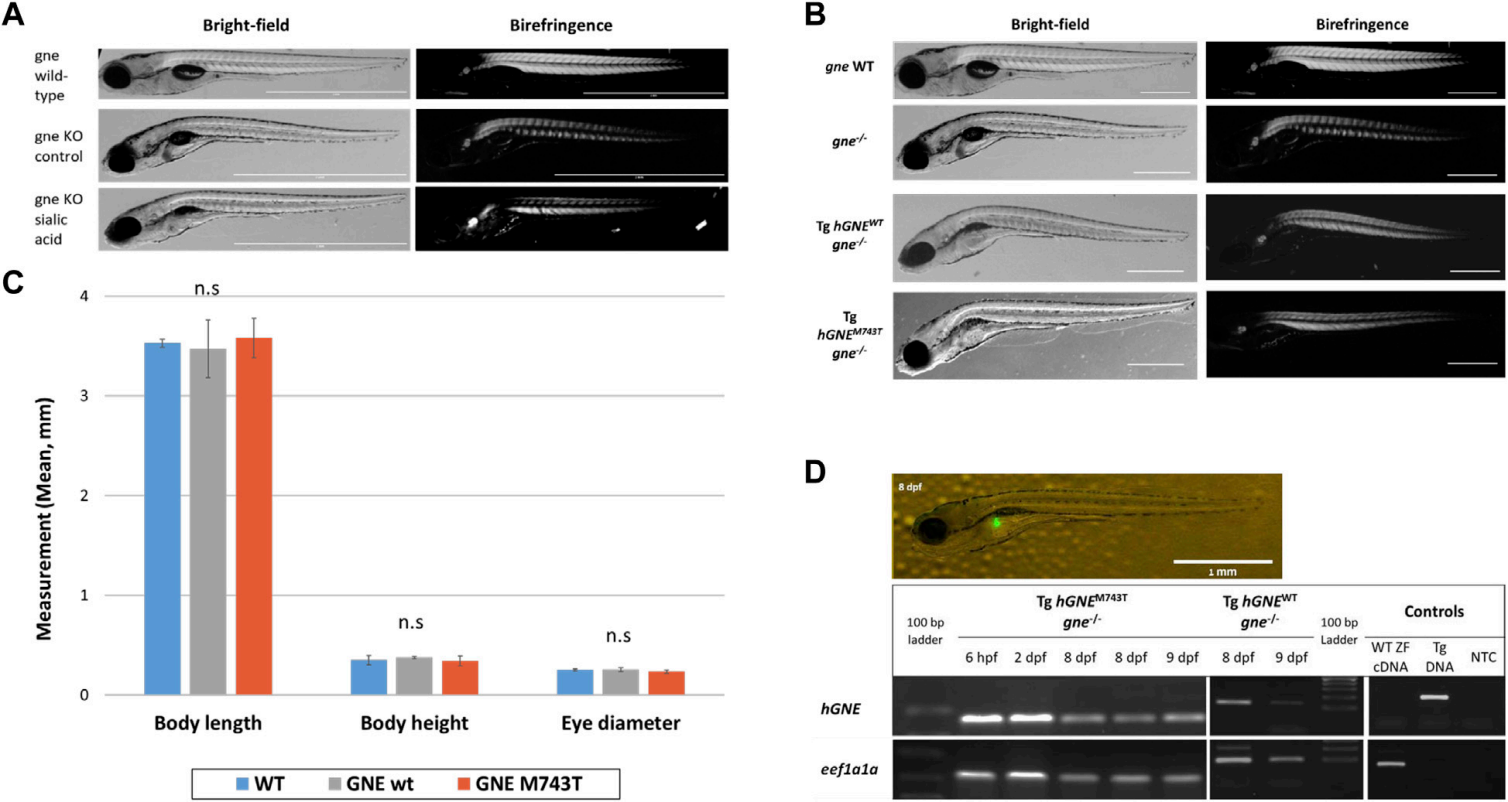
Exogenous sialic acid or *hGNE* transgene expression does not rescue the *gne* KO larvae. **(A)** Brightfield and birefringence representative images of 8 dpf larvae that participated in the sialic acid supplementation experiment: *gne* WT (top), *gne* KO (middle) and *gne* KO larvae with 800 μM sialic acid supplementation (bottom). The addition of sialic acid did not ameliorate the muscle phenotype, nor rescued *gne* KO larvae from mortality at 8–10 dpf. **(B)** Brightfield and birefringence images of GNE myopathy genetic model larvae display a phenotype similar to *gne* KO larvae at 8 dpf. **(C)** No significant differences were detected in morphologic measurements (body length, body height, eye diameter) between wild-type and *hGNE* ^M743T^ or *hGNE* ^WT^ transgenic KO models at 8 dpf. (Mann-Whitney Wilcoxon test, *p* > 0.05). Means of 10 larvae/variable/genotype and corresponding standard deviations are presented. **(D)** Transgene expression validation. Top: Fluorescent image of a representative 8 dpf Tg(*ins*:EGFP;*hGNE* ^M743T^ *;gne* ^−/−^) larva showing EGFP expression in the pancreas. Scale bar = 1 mm. Bottom: Agarose gel electrophoresis of RT-PCR amplification products of the *hGNE* cDNA and *eef1a1a*. cDNA samples from both *hGNE* ^M743T^ and *hGNE* ^WT^ transgenic KO models show the expected amplification in all tested time points. Non-transgenic WT zebrafish cDNA (WT ZF cDNA) and genomic DNA from transgenic fish (Tg DNA) served as controls for each target. (NTC) no template control.

To examine this possibility, we in-crossed heterozygote *gne* fish, collected the embryos at 1 cell stage, and transferred them to a Petri dish, with or without supplementation of sialic acid into the medium, at escalating concentrations from 200 to 800 µM. Larvae were monitored for 16 days post-fertilization. At 7 dpf, we noticed the appearance of the described *gne* KO phenotype in both the treated and untreated groups. All dead larvae were documented and sampled for genotyping throughout the experiment. At 16 dpf, all the surviving larvae were sampled and genotyped. No *gne* KO larva was found among them. All the larvae that died at 7–10 dpf showed a deflated swim bladder, curving of the body, reduction in muscle birefringence (Figure 8A), reduced movement, paralysis and decreased heart rate and were found to be *gne* KO. Therefore we conclude that sialic acid supplementation could not rescue *gne* KO fish.

### *hGNE* expression does not rescue *gne* KO larvae

In order to test the ability of human *GNE* cDNA to rescue the *gne* KO zebrafish from death, or ameliorate its symptoms, we generated a GNE Myopathy model by crossing the established transgenic line (described above, expressing the *hGNE* cDNA wild-type or M743T mutation under the control of zebrafish *gne* promoter) with the *gne* KO heterozygote fish to obtain an intermediate transgenic lineage. Those intermediate lines express the *hGNE* transgene and are heterozygote to the endogenic *gne* KO mutation. To obtain the genetic model, we crossed the intermediate transgenic lines again with the *gne* ^+/-^. Offspring larvae were monitored up to 16 dpf. At 2–3 dpf we screened the larvae for EGFP expression in the pancreas and separated the EGFP positive (with the *hGNE* transgene) and negative (without the *hGNE* transgene) larvae to different plates. At 7 dpf, we noticed the appearance of *gne* KO phenotype in the EGFP- group, as expected, but also in the EGFP + group, with both *hGNE* wild-type and *hGNE* M743T transgenic lines (Figure 8B).

At 16 dpf, all live and dead larvae were documented and sampled for genotyping. All transgenic larvae that died in the 8–10 dpf period were *gne* KO and presented the characteristic *gne* KO phenotype (deflated swim bladder, curving of the body axis, reduced movement, paralysis, and mortality). In contrast, all the larvae that survived after day 10 post fertilization were either *gne* ^+/+^or *gne* ^+/-^. Analysis of morphologic measurements of *hGNE* ^*M743T*^ and *hGNE* ^*WT*^ transgenic KO models did not show any statistically significant differences compared to wild-type larvae, at 8 dpf (Figure 8C). Therefore, we were not able to rescue *gne* deficiency with *hGNE* expression in this genetic model. One possible explanation is some kind of silencing of the *hGNE* transgene in these larvae, which prevents *hGNE* expression.

To examine whether the *hGNE* transgene was indeed expressed in these larvae, we tested them for *hGNE* expression by RT-PCR at 7, 8, and 9 dpf. The results show that the *hGNE* is indeed expressed in both WT and mutated transgenes in the genetic models (Figure 8D). From these findings, we conclude that despite the expression of its mRNA, the human *GNE* transgene cannot rescue the *gne* KO fish phenotype.

### Zebrafish *gne* KO transcriptomics

To molecularly characterize the consequences of *gne* deficiency, we analyzed the entire transcriptome by RNA sequencing of *gne* KO, heterozygotes and wild-type siblings. For this purpose, offsprings of *gne* ^+/-^ in-crosses were pooled (*n* = 20) by genotype and developmental stage (3, 5 and 7 dpf), and their total RNA was extracted for sequencing. The experiment was conducted on 3 biological replicates.

**FIGURE 9:**
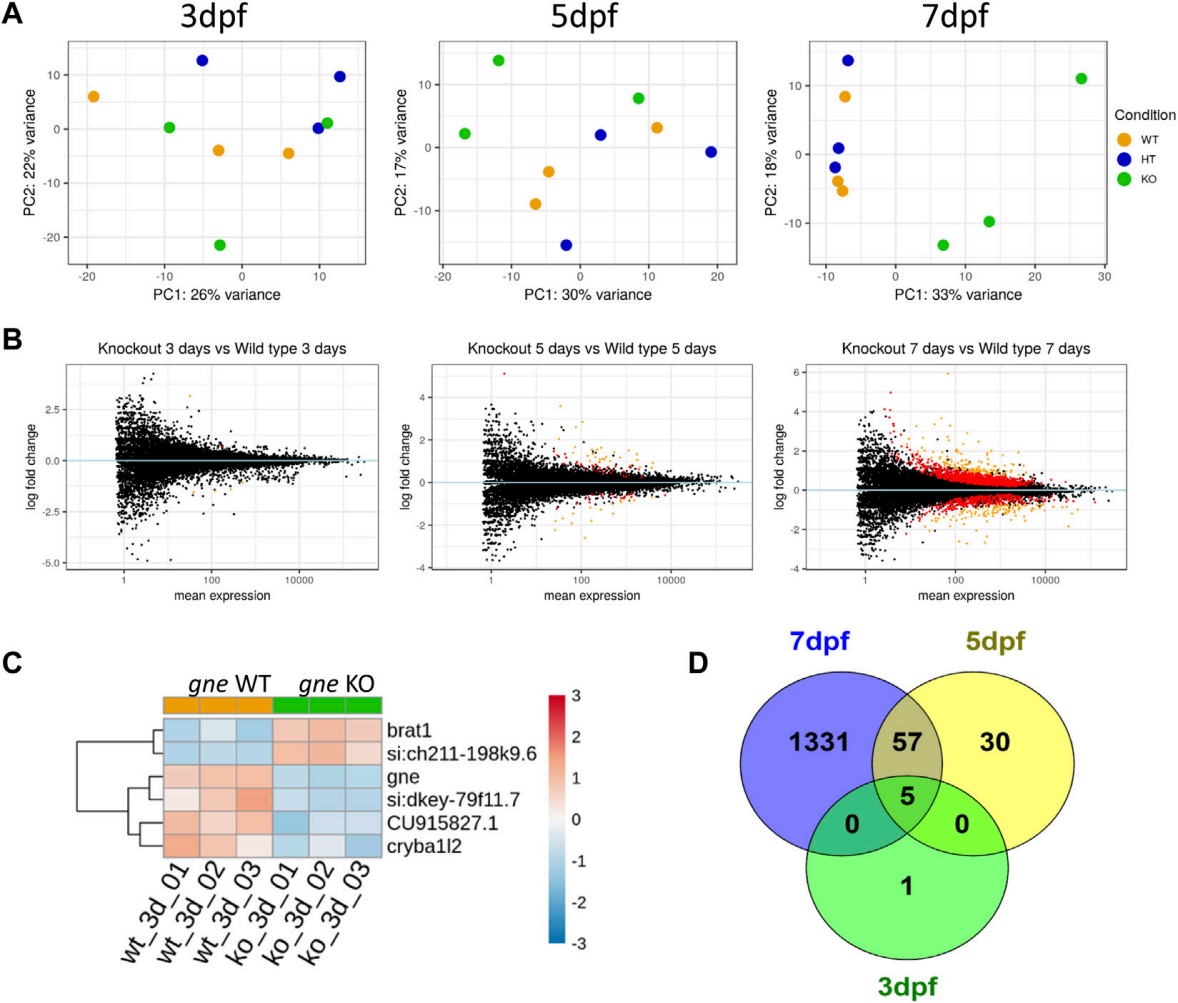
Transcriptomics analysis of *gne *KO larvae. **(A)** PCA plots of *gne* KO vs. *gne* WT at 3 dpf, 5 dpf and 7 dpf. *gne* KO cluster separates from gne WT and *gne* HT from 5 dpf onwards. **(B)** MA plots of *gne* KO *vs*. *gne* WT at 3, 5 and 7 dpf. Red and orange dots refer to significant differentially expressed genes, black dots are for non-significant genes. **(C)** Hierarchical classification of DE genes in *gne* WT *versus gne* KO at 3 dpf (3 biological replicates). The figure presents upregulated (red) and downregulated (blue) genes. (CU915827.1 = si:dkey 31e10.5). **(D)** Venn diagram indicating the number of significant differentially expressed genes among *gne* KO and *gne* WT siblings at 3 (green), 5 (yellow) and 7 (blue) dpf.

Differentiation between the genotypic states showed a developmental kinetics pattern as seen by PCA. At 3 dpf, the 3 genotypes cannot be separated, however at day 7 post-fertilization the genotype dictates the variance in gene expression (Figure 9A). This pattern indicates an early minor transcriptional response to *gne* loss at 3 dpf, which intensifies in the following days (Figures 9A,B). Since *gne* HT samples clustered together with the *gne* WT group, we decided to continue our analysis by comparing *gne* KO with *gne* WT only.

#### Differentially expressed genes

Differential expression analysis was performed with the DESeq2 package. Only 6 genes were significantly DE in *gne* KO larvae relative to *gne* WT siblings at 3 dpf (Figures 9C, D and Table 1). These 6 genes include *gne*, and are DE also at 5 and 7 dpf except for one, cryba1l2. At 5 dpf, 92 genes were significantly DE between the 2 genotypes, among them, 57 genes are also significantly DE at day 7 (Figure 9D). At 7 dpf 1331 additional genes were found to be significantly DE.

**TABLE 1:** Statistically Significant differentially expressed genes in *gne* KO versus *gne* WT zebrafish at 3 dpf.

Gene stable ID	Gene name	Biological process	log2Fold change	Adjusted *p*-value
ENSDARG00000099771	*gne*	Sialic acid Biosynthesis	−1.40	0.000
ENSDARG00000069804	*si:ch211-198k9.6*	Protein ubiquitination	3.18	0.000
ENSDARG00000092361	*si:dkey-79f11.7 (ifi44a5)*	Immune system	−1.49	0.001
ENSDARG00000062585	*brat1*	DNA damage	0.69	0.002
ENSDARG00000096564	*si:dkey-31e10.5*	Innate immune system	−1.54	0.007
ENSDARG00000014803	*cryba1l2*	Lens development	−1.07	0.076

The very small number (5) of differentially expressed genes at the early 3 dpf stage may give us a clue on the primary effects of *gne* deficiency. Three of these genes were downregulated; ***ifi44a5*** (si:dkey-79f11.7) is an interferon-induced protein involved in immune response; ***CU915827*** (si:dkey-31e10.5) is the zebrafish orthologue of the human *CD200* which encodes for a membrane glycoprotein involved in regulation of macrophage function. The third downregulated gene is ***cryb1l2***, involved in the eye lens development.

Two genes were upregulated, ***brat1***, which is involved in cellular response to DNA damage, and ***si:ch211-198k9.6***, which has a human orthologue, ***RTBBP6***, retinoblastoma tumor suppressor 6 binding protein gene, involved in cell cycle, mRNA processing and ubiquitination.

**FIGURE 10:**
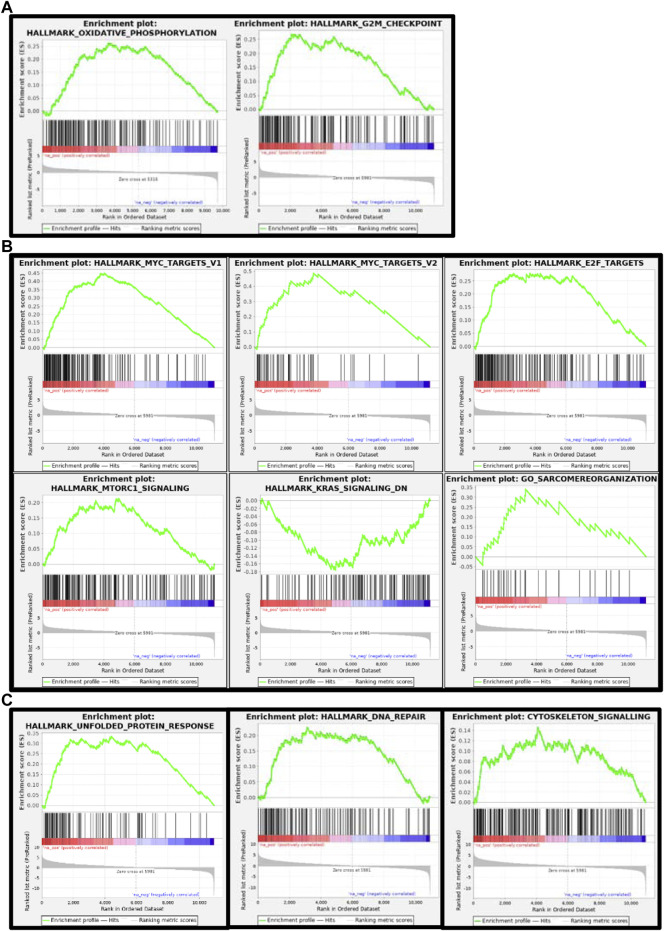
Gene set enrichment analysis (GSEA) plots of *gne* KO at 3 **(A)**, 5 **(B)** and 7 **(C)** dpf. GSEA plots show the enrichment score on the *y* axis for genes related to each gene set. Genes are ordered on the *x* axis according to their GSEA enrichment score. FDR adjusted *p*-value (<0.05).

To have better insights into the occurring trends at 3 dpf, we analyzed the data by Gene Set Enrichment Analysis (GSEA). Enriched pathways were related to oxidative phosphorylation and respiratory electron transport chain as well as to the cell cycle (Table 2 and Figure 10).

**TABLE 2:** Gene Set Enrichment Analysis (GSEA) of *gne* KO versus *gne* WT zebrafish at 3 dpf.

#	Gene set name	Enrichment score	Adjusted *p*-value
1	Hallmark_Oxidative_Phosphorylation	3.72794	0
2	Reactome_TCA_RESP_ELE	3.35290	0
3	Respiratory_Electron_Transport_Chain	3.10158	0
4	Hallmark_G2M_Checkpoint	2.22721	0.043

At 5 and 7 days dpf, most of the differentially expressed genes were upregulated. To gain some insights into the pathways involved at 5 and 7 dpf, we used GeneAnalytics and IPA enrichment softwares that gave overlapping results.

At 5 dpf, the most enriched pathways were the visual cycle (Tables 3, 4, 5), and energy metabolism. At 7 dpf, enriched pathways included protein ubiquitination, cell cycle and oxidative stress response. At the molecular function level, cell death and survival, gene expression, RNA damage and repair and RNA post-transcriptional modifications, were the enriched processes (as seen in Tables 6, 7, 8). GSEA analysis at these time points revealed additional enriched pathways related to cell cycle and muscle organization (Tables 5, 8 and Figure 10B). Several muscle-related genes were upregulated, such as *hsp90aa1* and *unc45b,* which are myosin-specific chaperones and *smyd1b* and *actb1*, which are involved in myofibril assembly.

**TABLE 3:** Gene Analytics top enriched pathways in *gne* KO versus *gne* WT zebrafish at 5 dpf.

#	Pathway	Score	Genes
1	The Visual Cycle I (vertebrates)	31.94	*CES2*, *RBP5*, *RPE65*, *RLBP1*, *STRA6*, *RDH5*
2	Melanin Biosynthesis	21.81	*OCA2*, *DCT*, *TYRP1*
3	Metabolism of Fat-soluble Vitamins	17.07	*RPE65*, *RLBP1*, *STRA6*, *APOB*, *RDH5*
4	Visual Cycle in Retinal Rods	13.57	*RBP5*, *RPE65*, *RLBP1*, *RDH5*
5	Glucose/Energy Metabolism	13.36	*ACSL1*, *IRS2*, *AHSG*, *FABP1*, *SLC34A2*, *ATF3*

**TABLE 4:** IPA top enriched pathways and biological processes in *gne* KO versus *gne* WT at 5 dpf.

#	Pathway	*p*-value	Pathway cover
1	The Visual Cycle	9.77E-08	20% (4/20)

#	Biological process	p-value range	#Genes
1	Lipid metabolism	1.75E-02—4.07E-08	22
2	Vitamin and Mineral metabolism	1.75E-02—1.20E-07	9

**TABLE 5:** Gene Set Enrichment Analysis (GSEA) of *gne* KO versus *gne* WT zebrafish at 5 dpf.

#	Gene set name	Enrichment score	Adjusted *p*-value
1	Hallmark_MYC_Targets_V1	6.50	0
2	Hallmark_Oxidative_Phosphorylation	6.46	0
3	Reactome_TCA_RESP_ELE	4.95	0
4	Hallmark_E2F_Targets	4.12	0
5	Hallmark_MYC_Targets_V2	4.05	0
6	Hallmark_G2M_Checkpoint	3.88	0
7	Respiratory_Electron_Transport_Chain	3.36	0
8	Hallmark_MTORC1_Signalling	3.30	0
9	Striated Muscle Contraction (Homo Sapiens)	3.29	0
10	Go_Filamentsliding	3.17	0
11	Hallmark_Fatty_Acid_Metabolism	2.86	0.002
12	Hallmark_Unfolded_Protein_Response	2.69	0.002
13	Reactome_Striated_Muscle_Contraction	2.61	0.002
14	Hallmark_DNA_Repair	2.61	0.003
15	P53	2.53	0.005
16	Go_Structural_Constituent_of_Muscle	2.42	0.012
17	NMD_Genes	2.28	0.044
18	Hallmark_Interferon_Alpha_Response	2.27	0.045
19	GO_Sarcomere_Organization	2.25	0.05
20	Hallmark_Kras_Signalling_DN	-2.24	0.054

**TABLE 6:** Gene Analytics top enriched pathways in *gne* KO versus *gne* WT zebrafish at 7 dpf.

#	Pathway	Score	Genes
1	Mitotic G1-G1/S Phases	105.31	*CKS1B*, *CDK2*, *RBBP4*, *MYC*
2	Ubiquitin –Proteasome Proteolysis	40.43	*PSMB2*, *UBA1*, *USP14*
3	Glucose metabolism	39.33	*SLC2A2*, *MDH1*, *UGP2*

**TABLE 7:** IPA top enriched pathways and biological processes in *gne* KO versus *gne* WT at 7 dpf.

#	Pathway	*p*-value	Pathway cover
1	Protein ubiquitination pathway	3.53E	19.8% (54/273)
2	Cell Cycle Control of chromosomal replication	3.02E-14	35.7% (20/56)
3	NRF2-mediated Oxidative stress respond	1.74E-08	14.3% (27/189)

#	Biological process	p-value range	#Genes
1	Cell Death and Survival	2.30E-06–5.99E-29	396

**TABLE 8:** Gene Set Enrichment Analysis (GSEA) of *gne* KO versus *gne* WT zebrafish at 7 dpf.

#	Gene set name	Enrichment score	Adjusted *p*-value
1	Hallmark_G2M_Checkpoint	5.69	0
2	Hallmark_MYC_Targets_V1	5.63	0
3	Hallmark_E2F_Targets	5.61	0
4	Hallmark_MTORC1_Signalling	5.13	0
5	Transport_Golgi	4.21	0
6	Hallmark_Unfolded_Protein_Response	3.66	0
7	Hallmark_MYC_Targets_V2	3.58	0
8	P53	3.28	0
9	Hallmark_DNA_Repair	2.97	0
10	Hallmark_Interferon_Gamma_Response	2.87	0
11	Hallmark_Interferon_Alpha_Response	2.84	0
12	Hallmark_Protein_Secretion	2.59	0.003
13	Cytoskeleton_Signalling	2.47	0.008

Notably, the DNA damage response process, initiated at 3 dpf as illustrated by *brat1* gene upregulation, was reinforced during development as this pathway was significantly enriched at 5 and 7 dpf.

Interestingly, the genes involved in the sialic acid-related pathways were not significantly differentially expressed in the KO larvae, except for *gne*. Its downregulated expression remained constant during the entire analyzed period (3-7 dpf).

### Validation of the transcriptomic data

**FIGURE 11:**
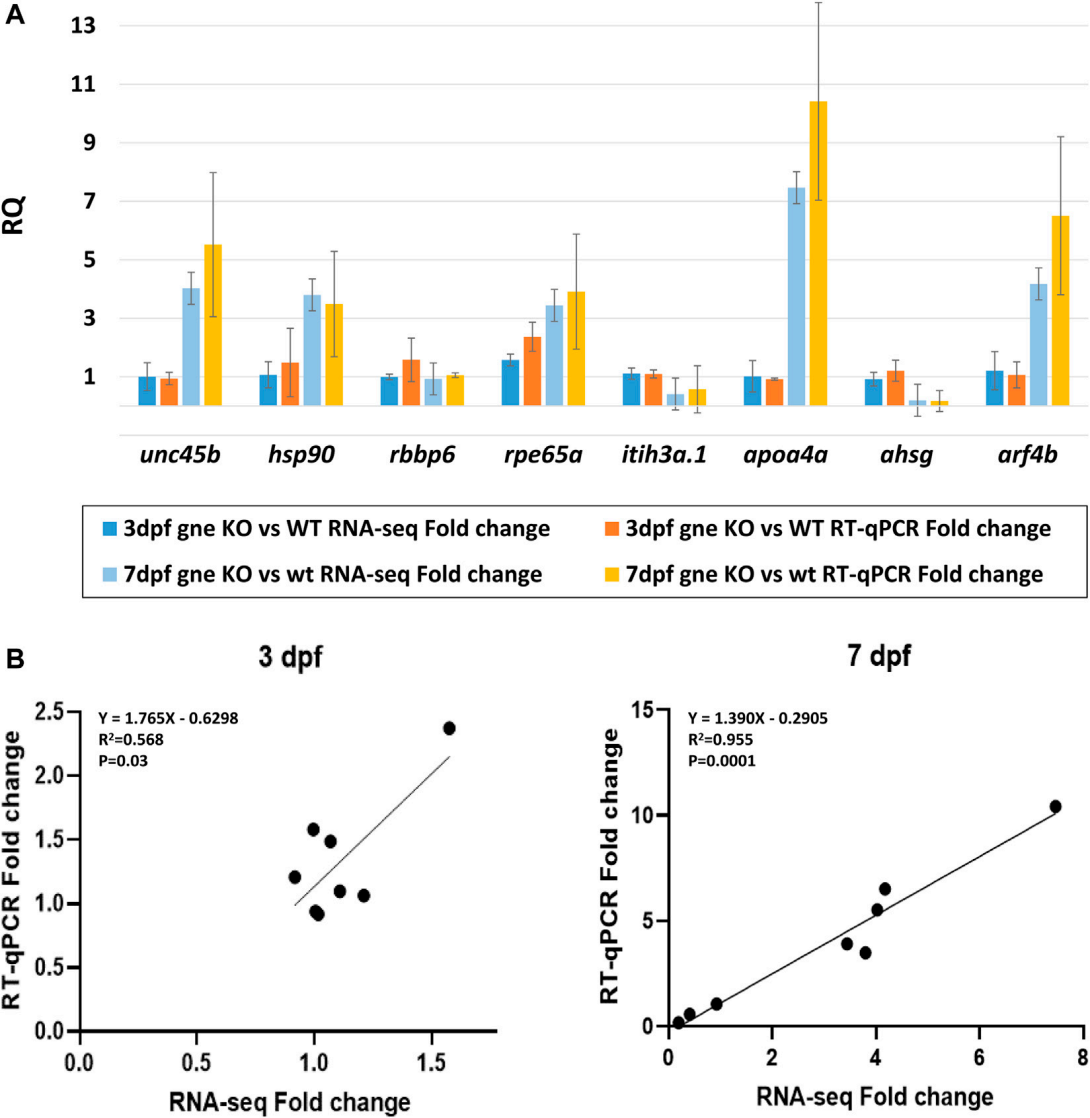
Validation of the transcriptomic data. **(A)** Comparison of the fold-change values between RNA-sequencing and RT-qPCR data for 8 chosen DE genes shows consistency at 3 dpf and 7 dpf samples (20 embryos/sample, 2 biological replicates). *Y* axis represents the mean RQ values, standard deviations are presented. **(B)** Gene expression correlation between RT-qPCR and RNA-seq fold-change at 3 dpf and 7 dpf. Linear regression equations, *R* ^2^ and *p* values are presented (Pearson’s correlation analysis).

To validate our transcriptomic results, we used TaqMan gene expression assays and performed RT-qPCR for 8 selected DE genes in *gne* KO and *gne* WT embryos RNA samples (20 embryos/sample) at 3 and 7 dpf, with 2 biological replicates. The *eef1a1a* gene was used for normalization. RT-qPCR expression of the 8 genes showed concordance with the RNA sequencing data. This was confirmed by the statistically significant correlation (Pearson’s correlation analysis) between the RT-qPCR and RNA sequencing data at both time points (Figures 11A, B).

## Discussion

The *GNE* gene and GNE Myopathy-associated mutations have been studied for two decades, but the mechanism in which *GNE* mutations lead to the development of a muscle pathology remains unclear. In this study we were able to investigate *GNE* function and dysfunction through genetic models we have established in zebrafish (*Danio rerio*). First, we have generated zebrafish expressing the human *GNE* gene, either wild-type or carrying the M743T mutation, the most frequent mutation in GNE Myopathy patients. These lineages show that concomitant expression of zebrafish and human *GNE*, even mutated, does not interfere with the development or lifespan of the fish. However, since no reliable anti-GNE antibody is available, we cannot determine whether gne protein was indeed overexpressed or that there is a threshold of *gne* expression accurately regulated that cannot be exceeded. Then, we developed a zebrafish lineage knocked out for the *gne* gene, generated by CRISPR/Cas9. Although the gRNAs used were designed and chosen not to target any exonic region in the zebrafish genome, in order to assess the validity of our CRISPR/Cas9 *gne* KO model, we minimized the off-targets risks by out-crossing the *gne* KO heterozygote fish with wild-type fish for 4 consecutive generations. Only the last generation was considered as the founder lineage for our studies. The fact that *Gne* KO in mice (Schwarzkopf et al., 2002) results in embryonic mortality at stage E8.5, together with the fact that to date, no human patient has been identified to carry 2 *GNE* null (−/−) mutations, allowed us to assume that the *gne* KO genotype might be lethal in zebrafish too. Indeed, *gne* is essential to zebrafish survival as well. However, the *gne* KO zebrafish show an overall normal development till day 7–8 post-fertilization, and then suddenly an abnormal phenotype appears, leading to death in the following 24 h. The lack of a detectable phenotype during the first week of life cannot be explained by the presence of maternally inherited *gne* mRNA, since it is identified only in the first few hours post-fertilization. Interestingly, maternally inherited *gne* KO mRNA is undetectable, as it most likely undergoes a rapid decay very shortly after synthesis. In contrast, the zygotic *gne* KO mRNA is persistently detectable, since although it also undergoes decay, it is expressed continuously.

The *gne* KO phenotype includes deflation of the swim bladder, reduced response to tactile stimuli, reduced heartbeat rate (probably a secondary effect, since it was only detected close to mortality, when the larva is completely paralyzed), and importantly for our purposes, impaired muscle organization. Indeed, birefringence assays suggested myofiber disorganization, as seen in other zebrafish models for nondystrophic myopathies (*Tmod4* ^*trg*^ mutants and *lmod3* ^*sa13018*^, Smith et al., 2013). This was confirmed by whole-mount immunostaining and histological sections. Whole mount F-actin staining of *gne* KO larvae at this stage revealed a wavy structure of both fast and slow muscle fibers in addition to their impaired organization. Whole-mount immunostaining of the slow muscle fibers showed that some of them detach from the myoseptum just prior to mortality. Further immunostaining of muscle-related proteins - actin, actinin and myomesin, at 8 dpf, exhibited normal localization, but the same muscle structural abnormalities. An important myofiber lack of organization as well as a wavy structure could be observed at least from day 5, although no motor dysfunction could be detected at this stage.

In addition to the muscle defects, *gne* KO zebrafish presented abnormal structures also in their eyes and brain. This is most likely related to the high expression of *gne* in these organs and confirms that sialic acid is essential for full development of these systems (Daya et al., 2014).

Interestingly, the *gne* KO phenotype could not be rescued by the continuous addition of sialic acid in zebrafish water, up to 16 days post fertilization. To note, studies by Van Karnebeek et al. (2016) and Wen et al. (2018) have shown that other disease phenotypes in zebrafish embryos could be rescued by exogenous sialic acid supplementation in the water. Therefore, it seems that the sudden appearance of phenotype and death is not a direct result of lack of sialic acid solely.

Most surprisingly though, transgenic expression of the human *GNE* cDNA could not rescue the *gne* KO zebrafish. We were unable to generate a viable *gne* ^−/−^;Tg *hGNE* ^*WT/M743T*^ zebrafish lineage. Those fish behave exactly as their *gne* KO counterparts, they all die around 8–10 dpf. Remarkably, a similar model in mice - an endogenous *Gne* KO carrying a *hGNE* transgene, either WT or carrying the M743T mutation - could not be generated either (Harazi, 2017). We have also tried to complement the *gne* KO larvae by injecting wt *gne* mRNA at a one-cell stage (data not shown). These experiments did not rescue the phenotype or lifespan of the fish, probably since the injected mRNA half-life is shorter than the period of time necessary for the *gne* KO larvae to develop a phenotype (∼7 dpf). From the successful generation of the zebrafish transgenic lineages, carrying the endogenous zebrafish *gne* and the human *GNE* cDNA, which develop normally, we know that the lack of rescue is not due to an absence of mRNA expression of the human transgene or due to toxicity. Several reasons could be considered to explain this finding. First, it could be that the human *GNE* gene lacks a specific zebrafish *gne* function, which is missing in the *gne* ^−/−^;Tg *hGNE* ^WT/M743T^ fish. Most likely, this function would not be the GNE known enzymatic activity, since the same human *GNE* cDNA, as was cloned in the transgene construct, was previously assayed *in vitro*, both for epimerase and kinase functions, and displayed both functions (Hinderlich et al., 2015). Another explanation could be that the human transgene cDNA used in these studies lacks potential regulatory regions, both at its 5' and 3' ends, since it begins from the first ATG and ends at the stop codon. The same construct was used in the mice studies mentioned just above. Hypothetically, these UTR sequences could precisely regulate the transcription/translation of the protein in a subtle manner to provide the additional essential function of GNE. It could also be that the amount of translated hGNE protein was too low, either as a result of a promoter lacking all regulatory elements or as a result of a human codon usage in the transgene, rather than a zebrafish codon usage. To examine these possibilities we are now trying to generate a zebrafish lineage with an endogenous M743T mutation in its *gne* gene, by the CRISPR/Cas9 methodology, so that no other region of the entire gene is disrupted.

To recognize more comprehensive processes taking place in the *gne* KO fish, RNA sequencing was applied at 3 developmental stages (3, 5, and 7 dpf), before *gne* KO mortality at 8–10 dpf. By this analysis, we could see that at 3 dpf there was almost no transcriptional difference between the different lineages. In contrast, at 5 and 7 dpf, the *gne* KO samples separated well from *gne* WT). These results correlate with the relatively late onset phenotype we observed for *gne* KO, which exhibits normal development at 3 dpf.

At 3 dpf, only 5 genes (in addition to *gne*) were significantly differentially expressed between both genotypes. Thus, most likely, the differential expression of these few genes has a critical impact on the upcoming phenotype at later stages of development of the *gne* KO zebrafish. Indeed, a progressively larger number of genes were differentially expressed at later stages. The minor transcriptional response at 3 dpf correlates with the phenotypic description of these larvae. Since the severe *gne* KO phenotype appears at 7-9 dpf, we assume that the earlier time-points of 3 and 5 dpf had more potential for detecting *gne* primary related mechanisms or functions which are affected in *gne* KO larvae. The relatively high number of DE genes at 7 dpf is probably the result of these earlier primary events, in addition to secondary related mechanisms involved in the upcoming severe *gne* KO phenotype.

Five of the 6 DE genes at 3 dpf remain DE at 5 and 7 dpf, keeping the same expression pattern. First, as expected, the *gne* gene is downregulated at all-time points in *gne* KO zebrafish. The downregulation of *ifi44a5* and *CU915827*, genes known to be related to immune response processes, might support an early change in immune response mechanisms in organisms lacking sialic acid, as previously suggested (Abeln et al., 2019; Briolat et al., 2014: Läubli and Varki, 2020; Wong et al., 2010). The genes *brat1* and *si:ch211-198k9.6* are upregulated at all 3 time points. *BRAT1* is known to be involved in the cellular response to DNA damage (Ertl et al., 2021). This result is particularly interesting since in a muscle cellular model knocked out for GNE that has been recently established in our laboratory, we also see this DNA damage/repair pathway affected (Ilouz et al., 2022). The human orthologue of *si:ch211-198k9.6*, is *RBBP6* (retinoblastoma tumor suppressor 6- binding protein), known to be involved in mRNA processing, cell cycle regulation, and ubiquitination activity (Xiao et al., 2019). This gene was also found to be associated with muscle myogenic differentiation in mice, and with muscle weakness in humans (Oikawa et al., 2011; Jones et al., 2021). The gene *cryba1l2*, which is significantly downregulated in *gne* KO only at 3 dpf, is known to be essential for normal lens development in zebrafish (Greiling et al., 2009) and could be related to the impaired eye morphology we observed in the *gne* KO zebrafish.

Since enrichment software is not applicable to such a small number of genes, we applied a Gene Set Enrichment Analysis (GSEA) to the dataset generated by RNA sequencing at 3 dpf. In this analysis, we could recognize upregulation in the processes of oxidative phosphorylation and energy metabolism pathways. Notably, involvement of these pathways was previously identified in our and other laboratories’ studies in *GNE* ^*M743T/*M743T^ mutated human muscle cells and tissues (Eisenberg et al., 2008; Cho et al., 2017), although the fold change was minimal.

Gene enrichment analysis (GeneAnalytics and IPA software) at 5 dpf revealed that the most enriched pathway is the visual cycle. This is in line with the fact that *gne* is strongly expressed in the retina (Daya et al., 2014) and with the impaired eye development observed in these fish. Interestingly, in addition to its broad role in muscle physiology, oxidative stress is also known to play a role in the visual cycle, including cataract formation (Verma, 1984). The oxidative stress response pathway, which was highly enriched in 7 dpf *gne* KO larvae, is consistent with previous studies that suggest an important role for oxidative stress in the pathology of several adult-onset muscle disorders (reviewed in Mosca et al., 2021), and specifically that GNE myopathy is associated with muscle oxidative stress in humans and mice (Cho et al., 2017).

GSEA at 5 dpf, in addition to what we already found at 3 dpf, revealed significant enrichment of upregulated functions related to the cell cycle, muscle function and organization, and a DNA damage/repair mechanism. The most enriched gene set was related to the function of the MYC transcription factor (Myc-proto-oncogene), which is known to regulate many major functions of growth, proliferation, death, differentiation, metabolism, self-renewal, and pluripotency (Carroll et al., 2018). A ChIP-seq analysis showed that the MYC transcription factor binds to the *GNE* promoter, and additional *in-silico* analysis predicted that *GNE* could interact with the MYC protein and that *GNE* M743T mutation could alter this interaction, and therefore might affect many potential pathways (Attri et al., 2021).

Gene enrichment analysis (GeneAnalytics and IPA software) at 7 dpf revealed many annotations, as expected from a large number of genes, but mostly reinforced the previously identified pathways. Notably, GSEA reveals novel insights on the involvement of *gne* in cell cycle, DNA repair, and more relevant to our studies, muscle-specific processes. Among the genes affected, many are relevant to muscle fiber organization. In particular, *hsp90aa1.1* is known to play a key role in myosin folding and sarcomere assembly. Its function could be regulated by post-translational modifications involving phosphorylation and acetylation, but no evidence for a role in glycosylation has been reported (Du et al., 2008). Similarly, *unc45b* is a chaperone required for the proper folding of myosin isoforms required for skeletal and cardiac muscle contraction (Wohlgemuth et al., 2007; Myhre et al., 2014). *Unc45b* interacts directly with skeletal muscle *myosin* and with *hsp90*. Interestingly, mutations in the myosin chaperone unc45b in zebrafish result in abnormal lens development and congenital cataract. In addition, *smyd1* is specifically expressed in muscle cells and interacts also with *hsp90*. Loss of function studies reveals that *smyd1* is required for sarcomere assembly in skeletal and cardiac muscles (Tan et al., 2006). In conclusion, lack of *gne* certainly impacts zebrafish muscle features.

The most novel insight from these studies is the involvement of cell cycle and DNA damage/repair processes in the *gne* KO zebrafish. This in turn could affect myogenesis and muscle regeneration processes. To note, despite the mutation in *gne*, the sialic acid related enzymatic gene set, upstream and downstream of the *gne* molecule, is not substantially altered in this experimental system. Previous studies have reported that sialic acid availability in mammal stem cells regulates the transcription of sialyltransferases, and absolute lack of sialic acid did upregulate few sialyltransferases in *Gne* knock-out stem cells (Milman Krentsis et al., 2011; Bork et al., 2017). Here we see only a slight change in mRNA expression of 1 among the 19 sialyltransferases detected in zebrafish, thus indicating that *gne* does not have a direct modulating effect on the expression of the enzymes in the sialic acid biosynthesis pathway as a whole, or that sialic acid is available to zebrafish from alternative sources. This hypothesis is further supported by the fact that long term sialic acid supplementation did not rescue the *gne* KO phenotype. Thus the known and new pathways shown to be affected in *gne* KO zebrafish reflect novel *gne* functions unrelated to sialic acid. In this context, we must emphasize that we have not been able to measure general or specific sialylation levels in the zebrafish due to technical difficulties of the current methods.

To conclude, we have established a *gne* KO zebrafish lineage and obtained new insights into *gne* functions. This is the only model where *gne* can be related to clear muscle defects, thus the only animal model relevant to GNE Myopathy to date. Further analysis of the newly described pathways could contribute to a better understanding of the role of GNE specifically in muscle. The elucidation of *gne* precise mechanism of action in these processes could be relevant to GNE Myopathy and allow the identification of novel therapeutic targets.

## Data Availability

The datasets presented in this study can be found in online repositories. The names of the repository/repositories and accession number(s) can be found below: GEO accession number: GSE207593 (NCBI tracking system #23084589) https://www.ncbi.nlm.nih.gov/geo/query/acc.cgi?acc=GSE207593.
