# Supplementary material for: Generation and characterization of a novel gne Knockout Model in Zebrafish
Source: Front Cell Dev Biol. 2022 Oct 24;10:976111. doi: 10.3389/fcell.2022.976111 (PMC9637792; doi:10.3389/fcell.2022.976111)

Supplementary Material

Table S1. Sequence of primers used in this study.

| **Primer name** | Sequence 5'-3' | Amplicon size |
| --- | --- | --- |
| *hGNE* exon 12 2132F | GGTTTCGGATTTGGTTGACC | 215 bp |
| *hGNE* exon 12 p.7193R | GCATTCTAGTTGTGGTTTGTCC |  |
| Zebrafish *gne* exon 3 28F | TGACTTTGACATCGGCTCCA | 399 bp |
| Zebrafish *gne* exon 3 427R | ATCGTCTCGTTTGTAGGCGG |  |
| Zebrafish *gne* wild-type specific F | CTTCCCGATGTCCTCC**AA** | 309 bp |
| Zebrafish *gne* mutation specific F | TTCCCGATGTCCTCC**TCT** |  |
| Human *GNE* cDNA 3F | CATGTGGCTAGGTGATGATGT | 142 bp |
| Human *GNE* cDNA 4R | GACTAGGGTCCGCTTGTTAAAT |  |
| Zebrafish *eef1a1b* 2F | GAAGCTGCAGAGATGGGAAA | 99 bp |
| Zebrafish *eef1a1b* 3R | CAGGGAGATATCGATGGTGATG |  |
| Zebrafish *gne* exons 2-3 junction F | TCATTGACGATTACGGAAACAC | 186 bp |
| Zebrafish *gne* exon 3 170R | CACCATGAACCAGCAGAATG |  |
| **TaqMan Probes** | Sequence 5'-3' | Fluorophore |
| Zebrafish *gne* wild-type specific | CCTCC**AACGTCTC** | JOE |
| Zebrafish *gne* mutation specific | CCTCC**TCTCG** | FAM |

**TaqMan gene expression assays IDs (Thermo-Fisher Scientific, US) by gene simbol.**


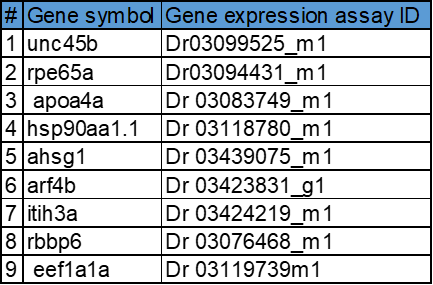


**Figure S1.** Mean Somite width measurements (somites 8-12) of 6 dpf *gne^–/–^* mutants did not identify a significant difference from wild-type larvae (Mann-Whitney Wilcoxon test, n=50, p>0.05)**.**

n.s

**Figure S2.** *gne* KO disrupts normal development of the eye and brain structures. Representative images of H&E stained transverse head sections, at the optic nerve level, of 5 dpf WT (top) and gne KO (bottom) larvae. Scale bars=200 μm (left images), 100 μm (right images).

**
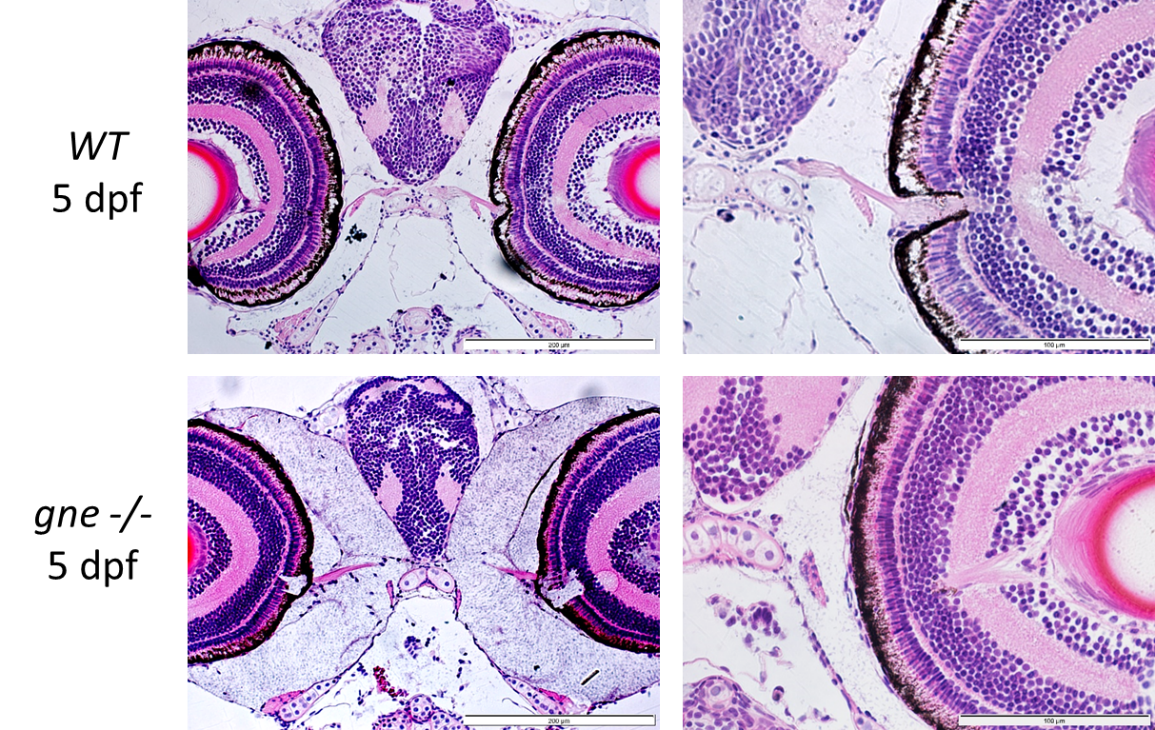
**

**Figure S3.** *gne* KO disrupts normal development of the eye and brain structures. Representative images of H&E stained transverse head sections, at the optic nerve level, of 8 dpf WT (top) and gne KO (bottom) larvae. Scale bars=100 μm.


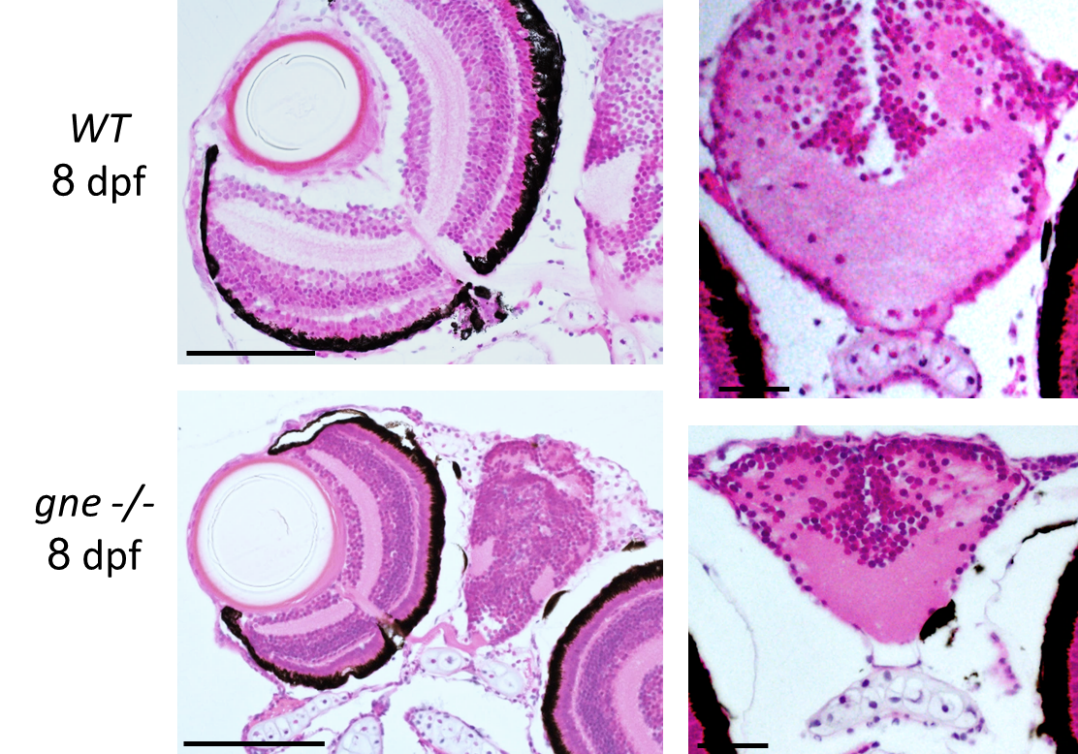

Supplement: Supplementary file 1 [file DataSheet1.docx]
